# Fetal Brain Development: Regulating Processes and Related Malformations

**DOI:** 10.3390/life12060809

**Published:** 2022-05-29

**Authors:** Zvi Leibovitz, Tally Lerman-Sagie, Leila Haddad

**Affiliations:** 1 Obstetrics-Gynecology Ultrasound Unit, Department of Obstetrics and Gynecology, Fetal Neurology Clinic, Wolfson Medical Center, Holon and Sackler School of Medicine, Tel-Aviv University, Tel-Aviv 5822012, Israel; sagie@wmc.gov.il; 2 Obstetrics-Gynecology Ultrasound Unit, Bnai-Zion Medical Center, Rappaport Faculty of Medicine, The Technion, Haifa 31048, Israel; leila.haddad-said@b-zion.org.il; 3 Pediatric Neurology Unit, Wolfson Medical Center, Holon and Sackler School of Medicine, Tel-Aviv University, Tel-Aviv 5822012, Israel

**Keywords:** CNS malformations, brain development, fetal neurology

## Abstract

This paper describes the contemporary state of knowledge regarding processes that regulate normal development of the embryonic–fetal central nervous system (CNS). The processes are described according to the developmental timetable: dorsal induction, ventral induction, neurogenesis, neuronal migration, post-migration neuronal development, and cortical organization. We review the current literature on CNS malformations associated with these regulating processes. We specifically address neural tube defects, holoprosencephaly, malformations of cortical development (including microcephaly, megalencephaly, lissencephaly, cobblestone malformations, gray matter heterotopia, and polymicrogyria), disorders of the corpus callosum, and posterior fossa malformations. Fetal ventriculomegaly, which frequently accompanies these disorders, is also reviewed. Each malformation is described with reference to the etiology, genetic causes, prenatal sonographic imaging, associated anomalies, differential diagnosis, complimentary diagnostic studies, clinical interventions, neurodevelopmental outcome, and life quality.

## 1. Introduction

Malformations of the central nervous system (CNS) occur in 14 out of 10,000 births. The true incidence is likely much higher (1/100) considering late-manifesting CNS anomalies [1]. Abnormal neurulation, telencephalic division, neuronal proliferation, migration, and cortical organization all play a role in the pathogenesis of CNS malformations. Abnormality in one or more of these developmental processes may be caused by specific chromosomal disorders or genetic mutations. Ischemia, hemorrhage, infection, or exposure to teratogens are all examples of insults to the fetal brain that can also result in CNS anomalies. Knowledge in this field has advanced dramatically in the last few decades due to developments in neuroimaging and genetics. The review references were mostly selected from original studies, clinical guidelines, textbooks, reviews, and meta-analyses. A better understanding of these malformations improves prenatal detection and allows for correct parental counseling. Pregnant women who have been diagnosed with fetal CNS anomalies should receive multidisciplinary consultation from specialists in fetal neuroimaging, pediatric neurology, and genetics.

## 2. Timetable of Human CNS Development

The development of the human central nervous system (CNS) is complex and governed by successive embryonic and fetal key processes: neural tube formation, separation of the telencephalic vesicle, neuronal and glial proliferation, neuronal migration, and cortical organization [2] (Table 1).

During the first six weeks of embryonic life, CNS development is governed by three sequential, overlapping phases: (1) gastrulation (formation of the three main germ layers, ectoderm, mesoderm, and endoderm); (2) dorsal induction (creation of the neural tube with three primitive vesicles: prosencephalon, mesencephalon, and rhombencephalon); and (3) ventral induction or telencephalization (separation of two cerebral hemispheres and formation of optic vesicles, olfactory bulbs, and corresponding facial structures) [3].

### 2.1. Dorsal Induction

**Figure 1:**
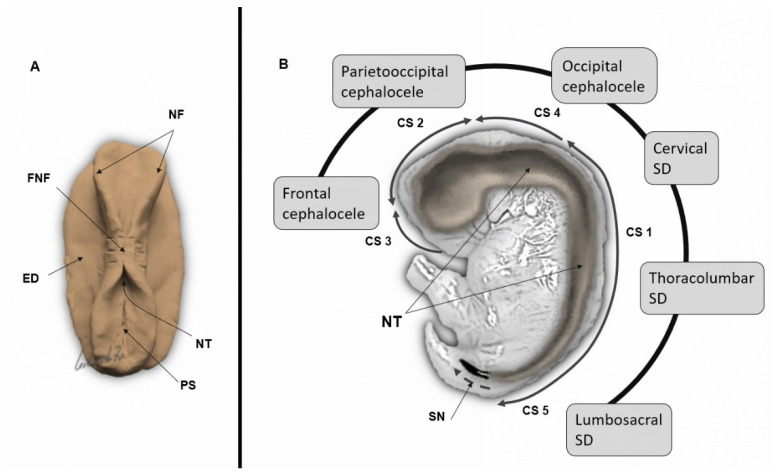
**Formation of the neural tube.** Legend: (**A**) An upper view of the embryonic disk at the 3rd postconceptional week. ED, the ectodermal surface of the embryonic disk; NF, neural folds; FNF, initial fusion of the neural folds, forming a neural tube (NT); PS, primitive streak. (**B**) A mid-sagittal section of a human embryo depicting closure sites (CS 1–5) of the neural tube (NT). Types of neural tube defects related to failed fusion of the neural folds at the specific closure sites (CS 1–5) are indicated in the text boxes. NT, neural tube; SD, spinal dysraphism; SN, secondary neurulation (the formation of the most distal part of the neural tube from the multipotential stem cells in the tail bud by longitudinal growth and canalization, indicated by the dashed arrow).

The neural tube begins to develop in the third postconceptional week, following the formation of the three main embryonic germ layers. At this stage, the embryo appears as an oval-shaped, three-layered disk. The CNS originates from a thickened and elongated median ectodermal zone, the neural plate. The lateral edges of the plate elevate, forming two neural folds divided by a central indentation, the neural groove. The upper boundaries of the neural folds fuse in the midline. Fusion begins at the mid-embryonic part and progresses in cephalic and caudal directions, forming the closed neural tube (primary neurulation) by the end of the fourth postconceptional week [4,5] (Figure 1A).

With closure of the neural tube, three primitive brain vesicles appear as adjacent dilatations along the rostral part of the neural tube: the prosencephalon (forebrain), mesencephalon (midbrain), and rhombencephalon (hindbrain), forming the anlage structures for the cerebrum, midbrain, and cerebellum, correspondingly (Figure 1B).

**Figure 2:**
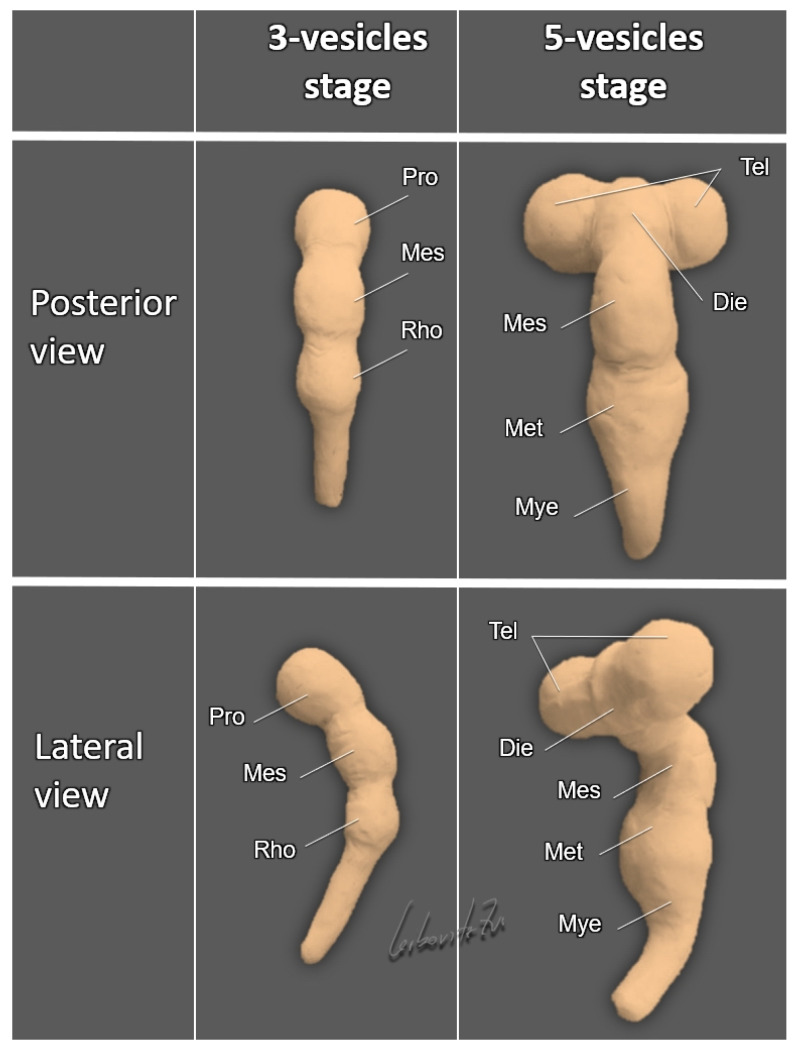
**Models of early neural tube development.** Legend: At the 3-vesicle stage, by the end of the fourth postconceptional week, three primitive brain vesicles are formed along the rostral part of the neural tube: the prosencephalon (Pro), mesencephalon (Mes), and rhombencephalon (Rho). At the 5-vesicle stage, by the seventh postconceptional week, the cephalic part of the prosencephalon rapidly grows and divides laterally into the two telencephalic vesicles (Tel) and a distinct medial diencephalic vesicle (Die). The rhombencephalon is divided into the metencephalon (Met) and the myelencephalon (Mye).

The primitive neural tube rapidly undergoes programmed spatial transformations in rostro-caudal, dorso-ventral, and lateral directions. By the end of the sixth postconceptional week, the tube develops three dorso-ventral flexures: the mesencephalic (at the level of the midbrain), the pontine (at the level of the rhombencephalon), and the cervical (at the junction between the rhombencephalon and the spinal cord) (Figure 2).

### 2.2. Ventral Induction

The cephalic part of the prosencephalon rapidly grows and divides laterally into the two telencephalic vesicles (the future cerebral hemispheres), whereas the caudal part of the prosencephalon forms a distinct diencephalic vesicle that gives rise to the optic vesicles and thalamic structures. The mesencephalon remains undivided, giving rise to the midbrain and the Sylvian aqueduct. The rhombencephalon divides into the metencephalon, from which the cerebellum and the pons will develop, and the myelencephalon, giving rise to the medulla oblongata (Figure 2). Such transformation of the embryonic CNS from a three- to a five-vesicle structure is completely evident at Carnegie stage 14, when the embryo is about 33 days old and is only 6 mm in length [5].

Patterning of the prosencephalon and mesencephalon is controlled by the homeobox genes *OTX1*, *EMX1*, and *EMX2*. Early neural tube development is dependent on the creation of two organizing centers: the anterior neural ridge, located at the junction of the neural tube with non-neural ectoderm, and the isthmus rhombocephali, between the hindbrain and midbrain. At both centers, FGF-8 and FGF-17 are the major signaling molecules that induce the expression of regulating genes. At the anterior neural ridge, they stimulate expression of *BF1*, which controls the development of the cerebral hemispheres, the basal telencephalon, and the retina. At the isthmic junction, *FGF-8* and *FGF-17* induce expression of the *EN1* and *EN2* homeobox genes responsible for the development of the dorsal mesencephalon (tectum) and cerebellum. *FGF-8* also induces *WNT1* expression, which interacts with *EN1* and *EN2* in early patterning of the midbrain and cerebellum. Formation and patterning of the isthmus are controlled by the balance of *GBX2* expression rostrally to the isthmic region, and *OTX2* caudally. Dorso-ventral patterning of the neural tube is regulated by *SHH* ventrally, and by *BMP4* and *BMP7* dorsally. In the myelencephalon, such patterning results in the development of the alar (dorso-lateral) and basal (ventro-lateral) plates, forming sensory and motor neurons, respectively [5].

### 2.3. Neurogenesis and Gliogenesis

Neurogenesis and gliogenesis refer to the proliferation and differentiation of neurons and glial cells from multipotent neural stem (progenitor) cells. This process begins at the late embryonic period and continues until the late second trimester. Neuronal cells are generated earlier than glial cells (oligodendrocytes and astrocytes) [6,7]. Neurogenesis and gliogenesis begin in the brain stem and spinal cord. Cerebral and cerebellar neurogenesis occur largely in the fetal period starting after 8 postconceptional weeks. Neurogenesis takes place primarily in the subependymal region of the lateral ventricles [8,9].

Initially, the neural stem cells dramatically expand their population by mitotic division. Over the course of late first and second trimesters, this massive cell proliferation in the ventricular and subventricular zones forms the germinal matrix, the tissue in which most cortical cells are produced [3,10]. During the early stages of neurogenesis, the radial glia cells in the ventricular and subventricular zones extend their long processes toward the pial surface on the external cortical border, providing an orthogonal glial scaffold for the neurons destined to migrate from the subventricular zone to the cortex [10,11]. At the seventh postconceptional week, the proliferating progenitor cells in the subventricular zone give rise to the first neurons [12,13]. The increase in population of neuronal cells in the human brain is closely related to proliferation and expansion of radial glia cells, which directs cortical growth and gyral formation [3]. Neuronal proliferation reaches its maximal rate at 15–16 postconceptional weeks, then gradually decreases by the end of the second trimester [2].

It is estimated that at least twice as many neurons as necessary are produced during the period of neuronal and glial proliferation. The excessive cells undergo apoptosis. Such programmed cell death occurs at the beginning of neurogenesis and during the second half of gestation, resulting in the elimination of excessive axons, dendrites, and synapses that may have less selective, diffuse, and inaccurate communications [12,14].

Neurogenesis is governed by numerous genes which regulate transcription, cell cycle progression, centrosome maturation (*MCPH1*, *CENPJ*, *CDK5RAP2*) [15], dynein binding, centrosome duplication (*NDE1*) [16], DNA repair (*MCPH1* [15], *PNKP* [17], and *PCNT* [18]), proliferative ability of the neural stem cells (*ASPM* and *STIL*) [19,20], and mitotic spindle formation (*WDR62* [21] and *NDE1* [22]). Mutations in these genes may result in primary microcephaly due to decreased neuronal and glial proliferation [23].

### 2.4. Neuronal Migration

Migration of neurons is essential for normal cortical development. Neural cells in the subventricular zone that complete their mitotic division program begin to move toward the outer zones of the developing brain. Active movement of the neurons is controlled by formation of actin filaments at the leading cellular edge, remodeling of the cytoskeleton by assembling and disassembling the microtubules, and appropriate function of the attaching proteins (integrins) on the cell surface.

The neurons migrate in radial and tangential fashions. Radially migrating neurons move centrifugally along glial processes that extend from the subependymal region towards the cortex [12]. Each migrating neuron is destined to reach its specific cortical layer and stay there for life. The pial membrane prevents neuronal over-migration outside the external brain surface [24]. The genes involved in regulation of the neuronal migration include *LIS1*, *ARX*, *DCX*, *RELN*, *TUBA1A*, *VLDLR*, *WDR62*, *NDE1*, *ACTB*, *FCMD*, *FKRP*, *POMT1*, and *POMT2* [25].

Tangentially migrating neurons emerge from ganglionic eminences and travel non-radially toward the cerebral cortex, guided by axons rather than radial glia [26] and by the interstitial guidance molecules from the Slit and semaphorin families [27]. Tangential migration provides most of the GABAergic interneurons of the cerebral cortex [28]. Most neurons move between the 12th and 20th weeks of gestation [5]. Neuronal migration occurs in subsequent waves. Each wave of migrating neurons travels past their predecessors, resulting in the later neurons being the closest to the outer surface, finally forming the six-layered cortex.

### 2.5. Post-Migration Neuronal Development and Cortical Organization

Post-migration neuronal development and cortical organization follows neuronal migration, starting at approximately 22–24 weeks of gestation. Cortical organization is a complex process that results in maturation of the six-layered cortex, outgrowth of axons and dendrites from cortical neurons, and development of inter-neuronal synapses, which continues into infancy. Cortical organization begins with formation of the subplate zone located below the developing cortical plate. Subplate neurons play a key role in the creation of the neocortical neural network. Axon guidance molecules excreted by subplate cells attract neural fibers ascending from the thalamus and descending from the cortex, leading to the formation of thalamo-cortical tracts. The subplate zone enables connectivity between different cortical areas in both hemispheres and guides the final migration to form the six-layered cortex. By 38 gestational weeks, the subplate zone disappears and is replaced by white matter [12].

The neuroglial cells (astrocytes and oligodendrocytes) proliferate and differentiate at the third trimester, whereas myelination of neural fibers occurs mostly after birth [2].

Abnormal progression of the embryonic and fetal CNS developmental processes can cause specific congenital CNS anomalies: abnormal dorsal induction leads to neural tube defects; disorders of ventral induction manifest with various types of holoprosencephaly; unbalanced neuronal proliferation and apoptosis causes microcephaly or megalencephaly; inadequate neuronal migration results in lissencephaly and heterotopia; and failed cortical organization presents as polymicrogyria or cortical dysplasia. Many of these malformations have underlying genetic variants and are associated with developmental delay, intellectual disability, and epilepsy (Table 1).

## 3. Anomalies of Dorsal Induction-Neural Tube Defects

Neural tube defects (NTD) are caused by abnormal embryonic dorsal induction, which regulates formation and closure of the neural tube between the 5th and 7th postmenstrual weeks. NTDs are common CNS abnormalities that affect 0.5–2% of pregnancies [29]. The NTD spectrum includes anencephaly, cephalocele, and spina bifida (Table 2).

According to the multisite closure model, fusion of the neural fold occurs at multiple locations along the developing neural tube [4] (Figure 1B). Fusion begins at the cervical region (closure site 1) and then spreads rostrally and caudally to the spinal region. Closure site 2 is located at the junction between the prosencephalon and mesencephalon, and closure site 3 ensues at the most rostral aspect of the forebrain. Although additional closure sites have been proposed in human embryos (closure site 4 at the caudal end of the hindbrain and closure site 5 at the lumbar region), only closures 1 and 3 were confirmed by histologic studies [30]. Specific NTD types can be explained by failed fusion of the neural tube at the corresponding closure sites (Figure 1B).

### 3.1. Cranial NTD

**Figure 3:**
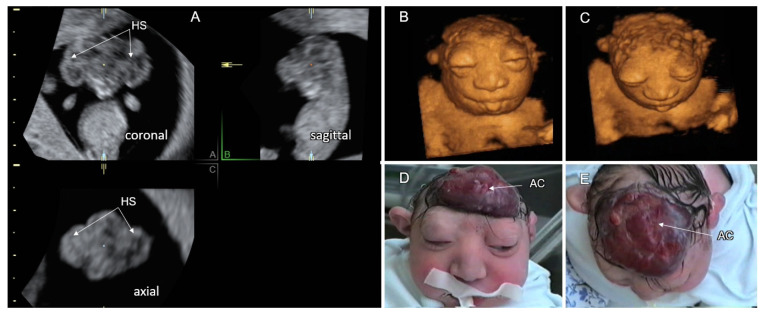
**Progression from acrania to anencephaly. Legend:** (**A**) A three-dimensional sonographic multiplanar reconstruction image demonstrating the coronal, mid-sagittal, and axial head planes of a fetus with acrania at 11 gestational weeks. Note the absent cranial vault and dysmorphic cerebral hemispheres (HS) floating in amniotic fluid above the base of the skull (exencephaly). (**B**,**C**) Three-dimensional sonographic surface render images of the fetal head at 27 gestational weeks. Note the flat, irregular area cerebrovasculosa (AC), a neural-vascular tissue remaining after traumatic destruction of the exposed cerebral hemispheres, the absent forehead, and protruding eyes. (**D**,**E**) Postpartum photographs corresponding to (**B**,**C**). The baby died 10 days after birth.

Cranial NTD may present with a total absence of the cranial vault (acrania) or a partial skull defect with herniated intracranial contents (cephalocele). The incidence of anencephaly is about 1 in 1000 births. Fetuses with acrania are frequently diagnosed at late first or early second trimester with a dysmorphic cerebrum, noncovered by the cranial vault (exencephaly). The diagnosis of exencephaly is often made on nuchal translucency scans by demonstration of the cerebral hemispheres floating in amniotic fluid above the base of the skull (the “Mickey Mouse” sign) [31,32]. The differential diagnosis between a large cephalocele with an extensive calvarial defect and exencephaly can be difficult. The exposed cerebral tissue undergoes traumatic destruction during later gestational weeks, resulting in anencephaly. The residual neural tissue includes only parts of the brainstem, the cranial nerves, and a variable amount of cerebellar tissue. The absence of the cranial vault and cerebral tissue on second and third trimester sonographic scans makes fetal anencephaly diagnosis simple. The calvarium and brain are replaced by a flat, irregular, vascular mass (area cerebrovasculosa), the forehead is absent, and the eyes protrude from the shallow orbits (the “frog’s face” sign). A typical progression of exencephaly in a fetus at 11 gestational weeks to anencephaly at 27 weeks is depicted in Figure 3. Anencephaly is a fatal malformation with a low risk of chromosomal anomalies and associated systemic malformations [33] (Table 2).

**Figure 4:**
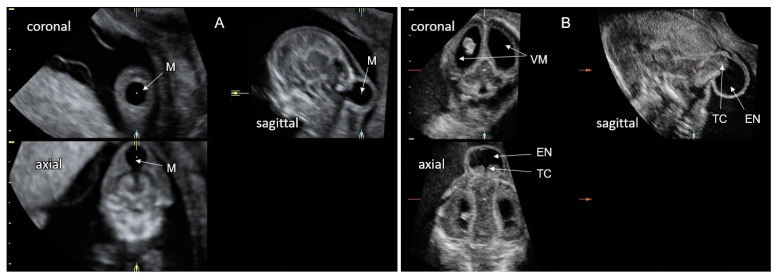
**Fetal posterior cephaloceles at the late first and early second trimesters.** Legend: (**A**,**B**) Three-dimensional sonographic multiplanar reconstruction images demonstrating the coronal, mid-sagittal, and axial head planes of two fetuses with posterior cephalocele. (**A**) An occipitocervical meningocele (M) without neural tissue inside the lesion at 12 gestational weeks. (**B**) Occipital encephalocele (EN), containing herniated tectum and cerebellum (TC) at 17gestational weeks; note the dilated lateral ventricles (VM).

Cranial meningocele is defined as the protrusion of meninges and cerebrospinal fluid through a skull defect without neural tissue inside the lesion; in an encephalocele, brain tissue is also herniated (Figure 4). Atretic cephalocele refers to a small midline subcutaneous head nodule that contains meninges and fibrous tissue, with or without brain tissue [33]. The incidence of cephalocele is 1–4 in 10,000 live births, ranking behind spina bifida and anencephaly. The occipital cephalocele is the most common subtype in Europe and the USA, while the frontal one is more common in Southeast Asia.

**Figure 5:**
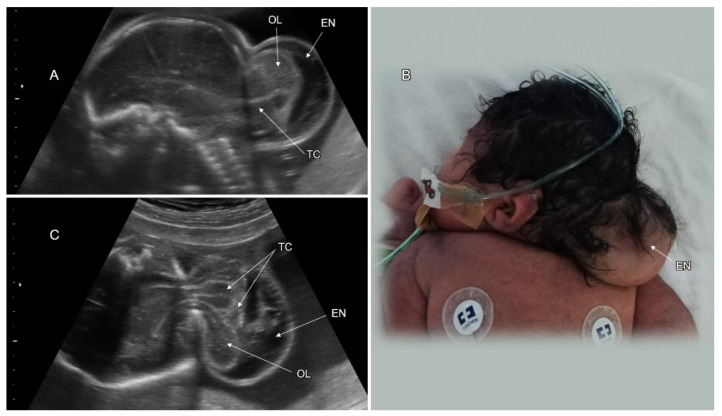
**Huge occipital encephalocele.** Legend: (**A**,**C**) Mid-sagittal and axial head sonograms of a fetus at 24 gestational weeks with a huge occipital encephalocele (EN) containing herniated occipital lobes (OL) and dysmorphic tectum and cerebellum (TC). (**B**) Postpartum photograph of the lesion; note the associated microcephaly. The baby died 3 days after birth.

**Figure 6:**
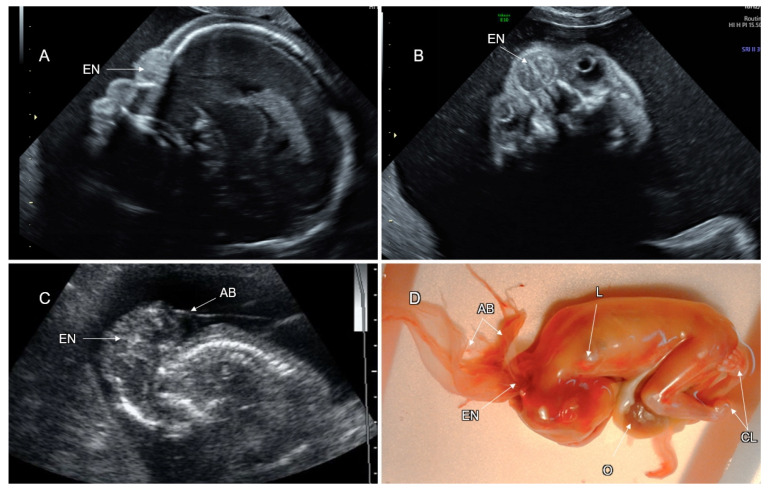
**Rare forms of cephalocele. Legend:** (**A**,**B**) Mid-sagittal and axial head sonograms of a fetus at 19 gestational weeks with a frontal encephalocele (EN) protruding through the cranial defect between the nasal and frontal bones. (**C**) Mid-sagittal sonograms of a fetus at 18 gestational weeks demonstrating a huge occipital encephalocele (EN); almost all the brain is located outside the calvarium connected to the amniotic band (AB); (**D**) Lateral photograph of the abortus (the exposed brain tissue was destroyed during abortion); note the amniotic bands (AB) connected to the cranial defect (EN) and associated malformations: the total absence of the left upper limb (L), omphalocele (O), and bilateral clubfoot (CL).

On fetal sonography, a cephalocele is recognized as a cystic formation protruding through a cranial vault defect. Demonstration of neural tissue within the cephalocele enables differentiation between meningoencephalocele and cranial meningocele (Figure 5). Multiplanar head scanning is recommended for optimal evaluation of cephalocele. The skull defect can be better demonstrated using the skeleton render mode of 3D ultrasound. Fetal head MRI can provide additional information regarding intra- and extracranial CNS findings, especially in the third trimester. Diagnosis of small or atretic cephaloceles can be facilitated by color Doppler demonstrating a falcine sinus or blood vessels running through the bony defect into the cephalocele [34]. Hypertelorism with asymmetric facial deformation in the orbital and nasal regions (Figure 6A,B) is a clue to the diagnosis of frontal cephaloceles. Cephaloceles are typically medial lesions. Lateral cranial defects may be caused by amniotic band syndrome presenting in association with omphalocele or limb reduction (Figure 6C,D).

More than two thirds of cephalocele cases may have additional CNS malformations (ventriculomegaly, microcephaly, agenesis of the corpus callosum, holoprosencephaly, cobblestone malformation, and spina bifida), cardiac, renal, and skeletal anomalies. Fetuses with cephalocele are at increased risk for chromosomal anomalies and syndromic conditions [35,36] (Table 2).

Although some soft tissue masses of the scalp (hemangioma, cystic hygroma, teratoma) may mimic cephalocele, none of them is characterized by an underlying cranial defect.

Prenatal diagnosis of a cephalocele requires thorough fetal scanning for associated structural anomalies and genetic investigations. Amniocentesis for chromosomal microarray (CMA) is indicated. Whole exome sequencing should be considered in cases with normal CMA results.

The postnatal outcome of babies with cephalocele correlates with the size of the lesion, herniation of neural tissue, hydrocephalus, and the presence of associated anomalies. About 15% of fetuses with encephalocele may die in utero and the mortality rate after birth ranges between 30–50% [35,36]. Cognitive impairment has been reported in more than 50% of patients with encephaloceles [33,37] (Table 2).

In fetuses with a high risk of unfavorable postnatal outcome, the option of pregnancy termination should be discussed with the parents. Delivery by cesarean section can decrease the risk of infection and trauma to the herniated brain tissue.

### 3.2. Spinal Dysraphism

Spina bifida or spinal dysraphism refers to a defect in fusion of the spinal portion of the neural tube, affecting 1 in 1000 newborns. Spinal dysraphism is classified as open (OSD) or closed (CSD) according to the absence or presence of skin tissue covering the defect, respectively. The mildest type of spinal dysraphism is spina bifida occulta, in which only the outer part of the affected vertebrae is not fused, whereas the neural spinal tissues, membranes, and skin are intact. Spinal meningocele is defined as a cystic bulge of the spinal canal membranes without neural tissues protruding through the defect in the vertebral arches. In myelomeningocele, the spinal cord and/or nerves extend into the lesion. The term “myelocele” is also used to describe spinal dysraphism without the bulging of neural tissue outside the defect [38]. Rachischisis is defined as a failed neural tube closure resulting in an exposed, uncovered spinal cord. Craniorachischisis is the most severe form of OSD in which the spinal cord and the brain are exposed.

Open spinal dysraphism is the commonest form of spina bifida (95%), manifesting as myelomeningocele or meningocele with a skin defect above the lesion. Closed spinal dysraphism is covered by intact skin and presents with or without a subcutaneous mass. CSD with a subcutaneous mass is due to lipomyelomeningocele or lipomeningocele. Closed spinal dysraphism without a subcutaneous mass is sub-classified into the simple forms (intradural lipoma, filar lipoma, tight filum terminale, persistent terminal ventricle, and dermal sinus) and the complex ones, related to defects of midline notochordal integration and segmental notochordal formation (dorsal enteric fistula, neurenteric cyst, diastematomyelia, caudal agenesis, and segmental spinal dysgenesis) [39].

**Figure 7:**
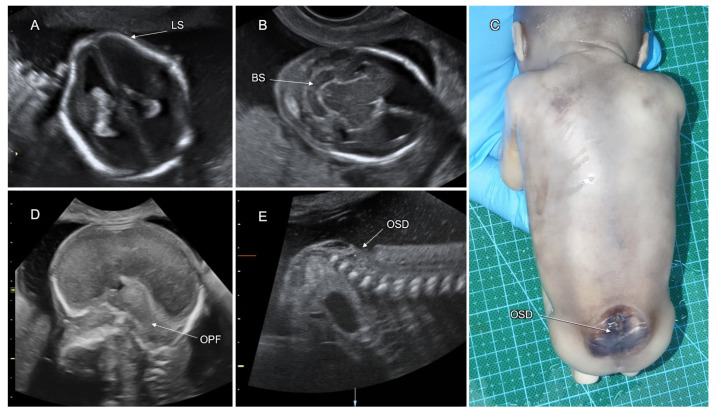
**Sonographic features of open spinal dysraphism in a fetus at 20 gestational weeks. Legend:** (**A**) Axial transventricular head plane, note the lemon-shaped fetal head (LS) due to scalloping of the frontal bones (the lemon sign). (**B**) Axial transcerebellar head plane, note the banana sign (BS): the curved cerebellar shape caused by the posterior displacement of the small brain towards the cranial vault within the obliterated cisterna magna. (**D**) Mid-sagittal head plane, note the small obliterated posterior fossa (OPF) with downward displacement of the brainstem, cerebellar tonsils, and vermis into the foramen magnum (Chiari-II malformation). (**E**) Mid-sagittal section through the lower spine. Note the open lumbosacral spinal dysraphism (OSD). (**C**) Posterior photograph of the abortus. Note that the lesion (OSD) is covered by a thin translucent membrane without normal skin.

**Figure 8:**
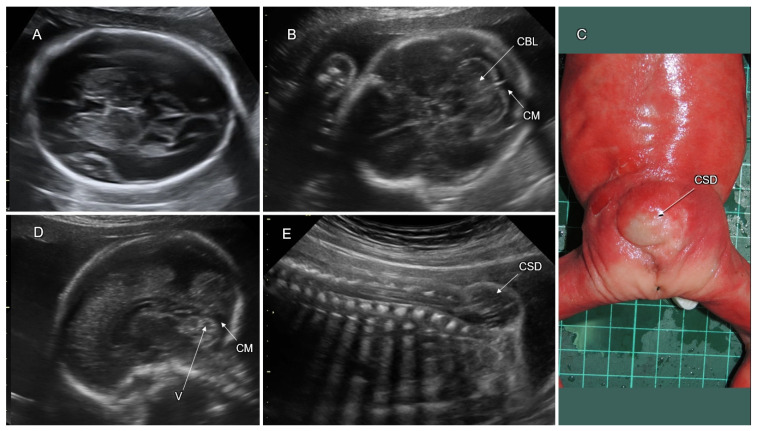
**Sonographic features of closed spinal dysraphism in a fetus at 25 gestational weeks Legend**: (**A**) Axial transthalamic head plane; note the normal shape of the fetal head. (**B**) Axial transcerebellar head plane; note the normal cerebellum (CBL) and cisterna magna (CM). (**D**) Mid-sagittal head plane; note the normal anatomy of the posterior fossa with a well-formed vermis (V) and a normal cisterna magna (CM). (**E**) Mid-sagittal section through the lower spine. Note the closed sacral spinal dysraphism (CSD) containing neural tissue (myelomeningocele). (**C**) Posterior photograph of the abortus. Note that the lesion (CSD) presents as a subcutaneous mass covered by normal skin.

Neuroimaging of OSD fetuses almost always shows obliterated supra- and infra-tentorial subarachnoid spaces, which is caused by cerebrospinal fluid (CSF) leakage through the spinal defect, leading to Chiari II malformation: downward displacement of the brainstem, cerebellar tonsils, and vermis into the foramen magnum (Figure 7). These changes may result in hydrocephalus, abnormalities of the corpus callosum, and absence of the cavum septi pellucidi (CSP). Contrary to OSD, CSD does not result in CSF leakage, and the brain anatomy is usually not affected [40] (Figure 8).

Sonographic diagnosis of fetal OSD at the second trimester is based on identification of the specific indirect features (the lemon and banana signs) and direct demonstration of the spinal defect [41]. The lemon sign describes the lemon-shaped fetal head in the axial plane due to scalloping of the frontal bones. The banana sign refers to the curved cerebellar shape caused by the posterior displacement of the small brain towards the cranial vault within the obliterated cisterna magna (Figure 7) [42].

Late-first-trimester sonography, routinely performed for nuchal translucency screening, was proposed by Chaoui et al. (2009) for early detection of OSD [43]. The authors used an abnormally small intracranial translucency for diagnosis of obliterated CSF spaces of the posterior fossa (specifically, the fourth ventricle) in fetuses with OSD. Although a first-trimester OSD diagnosis is possible, the detection rate is lower than at the second trimester [44]. Direct sonographic demonstration of fetal spinal dysraphism necessitates proper positioning of the fetal back during examination, as well as systematic multiplanar scanning, of the entire vertebral column, spinal cord, and overlying soft tissues. OSD can be recognized by discontinuity of the cutaneous contour with or without cystic formation, absence of vertebral arches, and widely separated lateral vertebral processes.

Diagnosis of fetal CSD without a subcutaneous mass is more difficult due to the absence of indirect signs and distortion of the medial spinal contour. However, a low position and posterior alignment of the conus medullaris can arise suspicion of a tethered cord and be a clue for detection of CSD.

Differential diagnosis of spinal dysraphism presenting as a bulging posterior medial lesion includes dermoid or epidermoid cyst, hemangioma, lipoma, and sacrococcygeal teratoma. In all of them, the vertebral arches beneath the lesion are intact.

OSD has multifactorial etiology. Most cases of spina bifida are of non-syndromic origin. In 2–16% of cases, spina bifida is associated with chromosomal abnormalities (predominantly trisomy 18) [45,46] and pathogenic CMA findings in 3.7% of cases [47]. Teratogenic causes of OSD include intake of antiepileptic drugs (i.e., valproic acid) and uncontrolled pregestational maternal diabetes mellitus [48].

In a small proportion of cases, the OSD can be a feature of morbid syndromes, including Jarcho–Levin, cerebrocostomandibular, Neu–Laxova, PHAVER, DiGeorge, SDAM (sacral defect with anterior meningocele), Czeizel–Losonci, Weissenbacher–Zweymüller, pentalogy of Cantrell, and OEIS (omphalocele–exstrophy–imperforate anus–spinal defects) complex [49] (Table 2). Many of these disorders can be confirmed by chromosomal microarray (CMA) and whole-exome sequencing (WES).

Periconceptional folic acid treatment can reduce NTD incidence by 60–70% [50]. Nevertheless, MTHFR gene mutations, which affect folate metabolism, have not been shown to contribute significantly to NTD prevalence [51]. The list of other candidate genes for OSD includes the planar cell polarity genes (VANGL1, CESLR1, and SCRIB), DNA repair and DNA methylation genes (APE1, XPD, and SOX18), retinol metabolism gene (ALDH1A2), oxidative stress genes (SOD1 and SOD2), and a cell adhesion molecule gene (NCAM1). Although more than 100 human genes have been studied for OSD [52], definite candidate genes have yet to be determined.

An increase in alpha-fetoprotein concentrations in maternal serum and amniotic fluid is a typical feature of open NTD and necessitates fetal neurosonography. Prenatal diagnosis of OSD should aim at detection of the associated CNS and systemic anomalies. The lower limbs should be evaluated for movements, presence of muscle atrophy, and deformation (clubfoot). Amniocentesis for CMA is recommended in view of the increased risk for genetic abnormalities.

Cesarean section should be considered to avoid trauma and infection of the OSD lesion during vaginal birth.

Fetuses with OSD are at significant risk for neurologic deficit and abnormal development. The risk is related to hydrocephalus secondary to the Chiari II malformation and dysgenesis of the spinal cord and nerves, resulting in paraplegia, clubfoot, and sphincter incontinence. On long-term follow-up, OSD is associated with moderate–severe ambulation deficit and sphincter malfunction in about 50% and 60%, correspondingly, and cognitive impairment (IQ < 70) in 19%. Progressive prenatal ventriculomegaly, a high level of spinal lesion, and the placement of a ventriculoperitoneal shunt are all risk factors for poor motor function [40] (Table 2).

In utero OSD surgical repair was initially reported to improve postnatal motor and mental functional scores [53]. However, in a recent systematic review and meta-analysis on the neurodevelopmental outcome of children with open spina bifida, the risk of neurodevelopmental impairment was similar in those who were repaired prenatally and postnatally, although the cases operated in utero had an increased risk of prematurity [54].

In the United States, approximately 90% of babies born with OSD survive the first year of life, and 75–80% survive to early adulthood [55,56]. The outcome of CSD is significantly better than that of OSD. The majority of postnatally diagnosed CDS cases are incidentally detected as having occult spina bifida without a subcutaneous mass.

## 4. Holoprosencephaly—A Disorder of Ventral Induction

Holoprosencephaly (HPE) is the main anomaly of ventral induction. Prosencephalic development follows three overlapping sequential phases: formation, cleavage, and midline formation [57] (Figure 2). HPE results from a disturbance of normal cleavage of the prosencephalon into two telencephalic vesicles between the sixth and ninth postmenstrual weeks, forming the cerebral hemispheres. Rudimental formation of the prosencephalon is a very rare anomaly resulting in aprosencephaly (absence of telencephalon and diencephalon) or atelencephaly (only the telencephalon is absent) [58]. Other defects of ventral induction are related to abnormal formation of the prosencephalic midline, presenting with agenesis of the septum pellucidum, septo-optic dysplasia, and agenesis of the corpus callosum. HPE is frequently associated with midline facial defects [59]. HPE is the most common developmental disorder of the forebrain and is attributed to numerous genetic and environmental factors. HPE occurs in about 1/250 of conceptuses. Because of the high incidence of associated anomalies, only 1/10,000 reaches livebirth [59].

In 1963, DeMyer proposed the most accepted classification of HPE. He described alobar, semilobar, and lobar HPE forms based on the degree of failure of prosencephalic cleavage [60] (Table 3).

**Table 3:** Classification of holoprosencephaly.

Type of Holoprosencephaly	Main Features (See Figure 9)
**Alobar holoprosencephaly**	A complete lack of separation of the cerebral hemispheres (a single-midline ventricle).
**Semilobar holoprosencephaly**	Only anterior lobes fail to separate.
**Lobar holoprosencephaly**	Only the most rostral-inferior parts of the frontal lobes are fused.
**Middle interhemispheric variant (syntelencephaly)**	The posterior frontal and parietal lobes fail to separate. The anterior and occipital cerebral aspects are divided.
**Septopreoptic holoprosencephaly** **(minimal form)**	A mild subtype of lobar HPE, midline fusion restricted to the septal region or preoptic region of the telencephalon.
**Microform holoprosencephaly**	Only subtle facial midline features: hypotelorism, single maxillary central incisor, and narrowing of the nasal pyriform aperture.

### 4.1. Alobar HPE

**Figure 9:**
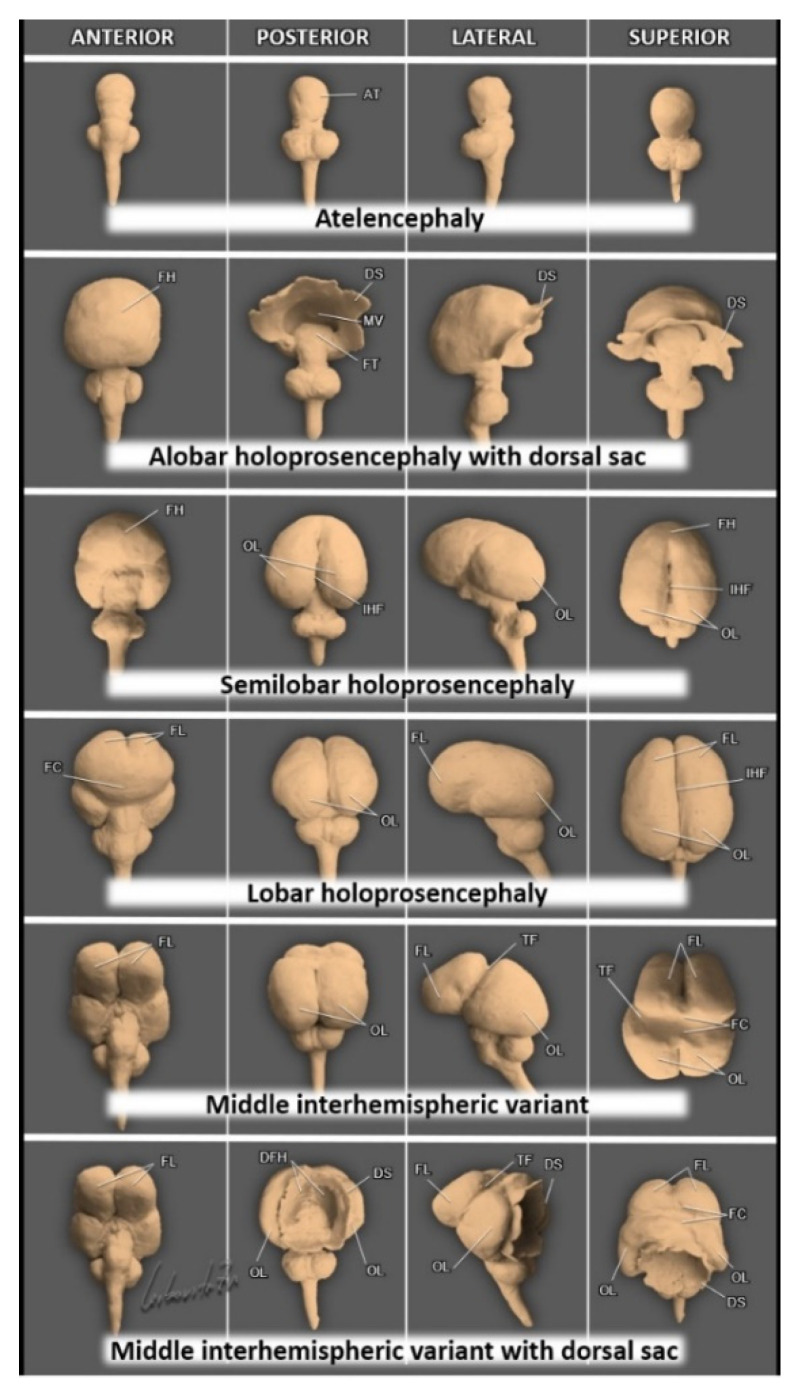
**Models of the holoprosencephaly spectrum. Legend:** Atelencephaly: rudimentally formed fused telencephalons (AT). Alobar holoprosencephaly: completely fused hemispheres (FH) lacking any separation with a single undivided midline ventricle (MV). Note the fused thalami (FT) and the ruptured dorsal sac (DS). Semilobar holoprosencephaly: the cerebral hemispheres are fused anteriorly (FH); the occipital lobes (OL) are separated by the interhemispheric fissure (IHF). Lobar holoprosencephaly: the cerebral hemispheres are almost completely separated. Fusion of the hemispheres occurs only in the rostral-inferior frontal cortex (FC). FL, divided frontal lobes above the fused cortex. Middle interhemispheric variant (MIH): the hemispheric fusion (FC) takes place only between the posterior frontal and parietal lobes. Note the abnormal transverse fissure across the midline (TF). Middle interhemispheric variant (MIH) with dorsal sac: the hemispheres are fused (FC) only between the posterior frontal and parietal lobes. Note the abnormal transverse fissure across the midline (TF), the divided frontal horns (DFN) of the lateral ventricles, and the ruptured dorsal sac (DS) separating small occipital lobes (OL).

**Figure 10:**
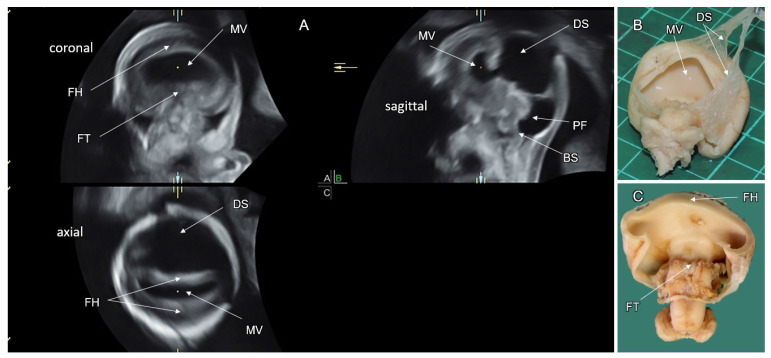
**Sonographic features of alobar holoprosencephaly in a fetus at 18 gestational weeks. Legend:** (**A**) A three-dimensional sonographic multiplanar reconstruction image demonstrating the coronal, mid-sagittal, and axial head planes. Note complete fusion of the hemispheres (FH), the undivided monoventricle (MV), and the thalamic fusion (FT), resulting in obstruction of the CSF flow into the aqueduct of Sylvius and the formation of a large dorsal cyst (DS). (**B**,**C**) Posterior photographs of the autopsy brain specimens, demonstrating the monoventricle, fused hemispheres (FH) and thalami (FT); note the ruptured membranes of the dorsal sac (DS), see also Figure 9.

In alobar HPE (no cleavage), a single monoventricular forebrain is formed. The interhemispheric fissure and the midline structures (the falx cerebri, the corpus callosum, and the cavum septi pellucidi) are absent. Alobar HPE is typically associated with fusion of the basal ganglia, thalami, and choroid plexuses. Thalamic fusion may result in obliteration of the third ventricle and obstruction of CSF flow into the aqueduct of Sylvius, leading to formation of a large dorsal cyst [57] (Figure 9). In the mid-sagittal head view, alobar HPE can appear in three distinct forms according to the amount of brain parenchyma around the monoventricle: a “ball”-shape (the monoventricle is completely enclosed by cerebral parenchyma); a “cup” (the parenchyma encloses the monoventricle at the anterior aspect), and a “pancake” (only small flattened inferiorly located cerebral parenchyma is present). Alobar HPE is differentiated from other HPE types by a monoventricle surrounded by the undivided brain parenchyma and fused thalami (Figure 10). Differentiation of hydranencephaly and severe hydrocephalus from alobar HPE is based on normal thalamic cleavage and visualization of the interhemispheric fissure and falx cerebri [59]. Alobar HPE is associated with the most severe facial malformations, affecting the development of the eyes, nose, upper lip, and palate. The eyes and optic nerves, and olfactory bulbs and tracts may be separated, fused, or absent [61]. The ophthalmologic features include cyclopia (single central eye), synophthalmia (two fused eyes in the midline), hypotelorism, and colobomas. Nasal defects can manifest as a lack of a nose with a proboscis (ethmocephaly) or as a small nose with a single nostril (cebocephaly). The oral anomalies of alobar HPE may manifest with a midline cleft lip and/or palate [59].

### 4.2. Semilobar HPE

In semilobar HPE (partial hemispheric cleavage), the frontal lobes are fused, but the parieto-occipital regions are divided by the interhemispheric fissure and falx cerebri [60] (Figure 9). The CSP is absent. The posterior part of the corpus callosum may form. The thalami are usually fused, and a dorsal cyst is common [61]. Facial malformations are usually milder compared to alobar HPE [59].

### 4.3. Lobar HPE

**Figure 11:**
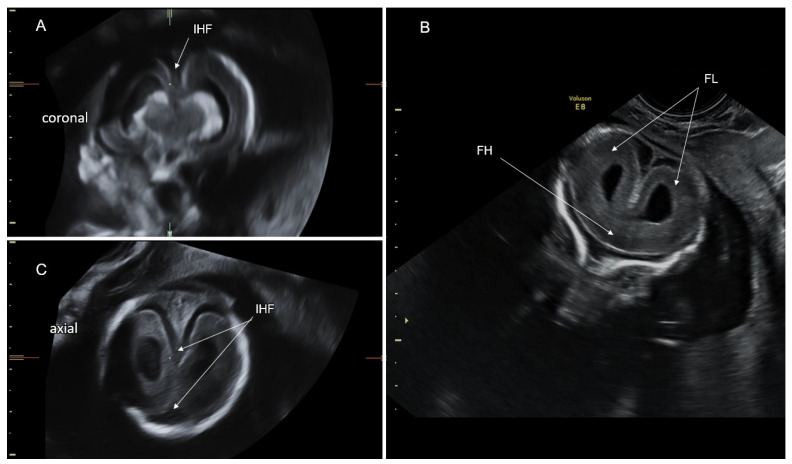
Sonographic features of lobar holoprosencephaly in a fetus at 19 gestational weeks. Legend: (**A**,**C**) Axial and coronal head planes. The cerebral hemispheres are separated along the upper aspect of the interhemispheric fissure (IHF). (**B**) Coronal transfrontal head plane. Fusion of the hemispheres occurs only in the rostral-inferior frontal cortex (FC). The frontal lobes (FL) are divided above the fused cortex. Note complete fusion of the hemispheres (FH), the undivided monoventricle (MV), and the thalamic fusion (FT), resulting in obstruction of the CSF flow into the aqueduct of Sylvius and the formation of a large dorsal cyst (DS). (**B**,**C**) Posterior photographs of the autopsy brain specimens, demonstrating the monoventricle, fused hemispheres (FH) and thalami (FT); note the ruptured membranes of the dorsal sac (DS), see also Figure 9.

In lobar HPE (almost complete hemispheric cleavage), only the inferior frontal lobes are fused, and the majority of the interhemispheric fissure and falx cerebri are well-formed along the midline. (Figure 9). The thalami are usually separated. These characteristics make diagnosing lobar HPE more difficult than alobar and semilobar HPE (Figure 11).

In fetuses with lobar HPE, the cavum septi pellucidi is typically non-visualized and the fornices are fused, but the corpus callosum may be normal or partially formed [61,62]. A color Doppler study of the anterior cerebral arteries, which deviate towards the anterior calvarial border due to frontal lobe fusion (a “snake under the skull” sign), can aid in the diagnosis of lobar HPE [63].

### 4.4. Middle Interhemispheric Variant

In 1993, Barkovich et al. [64] described a distinct form of HPE, the middle interhemispheric variant (MIH). The anterior and occipital hemispheric regions are separated in the MIH, while the posterior frontal and parietal lobes are fused. (Figure 9). The body of the corpus callosum may be absent in the MIH, but the genu and splenium are preserved. The Sylvian fissures are often vertically oriented and abnormally connected across the midline. Cortical dysplasia and subcortical heterotopia are common. A dorsal cyst appears in 25–40% of MIH cases. MIH is usually not associated with midline craniofacial anomalies [65] (Figure 9). Cortical continuity between the hemispheres restricted to the posterior frontal and parietal areas is a diagnostic feature of MIH.

### 4.5. Milder and Minimal Forms of HPE

Milder and minimal HPE forms may be associated with septo-optic dysplasia and nonseparation of the preoptic area, respectively [61,66]. A microform HPE is characterized only by facial features (hypotelorism and single maxillary central incisor) and normal brain development [57] (Table 3).

By the end of the first trimester of pregnancy, alobar and semilobar HPE can be reliably diagnosed. The diagnostic sonographic features include complete or partial absence of the interhemispheric fissure, distorted appearance of the choroid plexuses in the axial transventricular plane (an abnormal “butterfly sign”), fused thalami, median facial anomalies, and small biparietal diameter [67].

Prenatally diagnosed HPE is frequently associated with chromosomal abnormalities and syndromes. Aneuploidy has been reported in as many as 55% of fetuses diagnosed with HPE (mainly trisomy 13, trisomy 18, and triploidy) [59]. The prevalence of abnormal chromosomal microarray in syndromic HPE reaches 22% [68].

CHARGE (*CHD7*), Pallister Hall (*GLI3*), Smith Lemli Opitz (*DHCR7*), Rubinstein–Taybi (*CREBBP*), Velocardiofacial (*TBX1*), Meckel, Lambotte, and Aicardi syndromes are all genetic syndromes associated with euploid HPE [69].

Mutations in *SHH*, *ZIC2*, *SIX3*, *TGIF*, *GLI2*, *FGF8*, and *FGFR1* genes can be detected in about 20% of non-syndromic isolated HPE cases with a normal karyotype. *SHH* mutations are the most common cause of this group. They manifest with variable penetrance and lead to classic HPE forms in only 50% of the patients. Parental hypotelorism or a single maxillary central incisor can be the only indication of the presence of a familial *SHH* mutation [69].

Nongenetic etiologies of HPE include uncontrolled pregestational maternal diabetes mellitus, maternal alcoholism, and embryo exposure to retinoic acid.

HPE is associated with high postnatal mortality. Only a small proportion of children with HPE survive into adulthood. Survival is better in non-syndromic, euploid HPE patients. The severity of brain and facial defects is inversely correlated with survival. Almost all children with classic forms of HPE and MIH have abnormal neurodevelopment, which correlates with the severity of the CNS findings. Typically, alobar HPE results in profound global developmental impairment. Patients with semilobar, lobar HPE, and MIH can develop variable degrees of motor and speech skills [68].

Common medical problems in HPE survivors include hydrocephalus, epilepsy, motor and coordination impairment, pulmonary problems due to the risk of aspiration, gastrointestinal disorders, oromotor dysfunction, poor feeding and nutrition, endocrine dysfunction (central diabetes insipidus), hypothalamic dysfunction (abnormal sleep–wake cycles, temperature regulation, and impaired thirst control), ophthalmologic problems (visual impairment and strabismus), and craniofacial-related complications (facial cleft and upper airway obstruction) [69].

## 5. Disorders of the Corpus Callosum (DCC)

**Figure 12:**
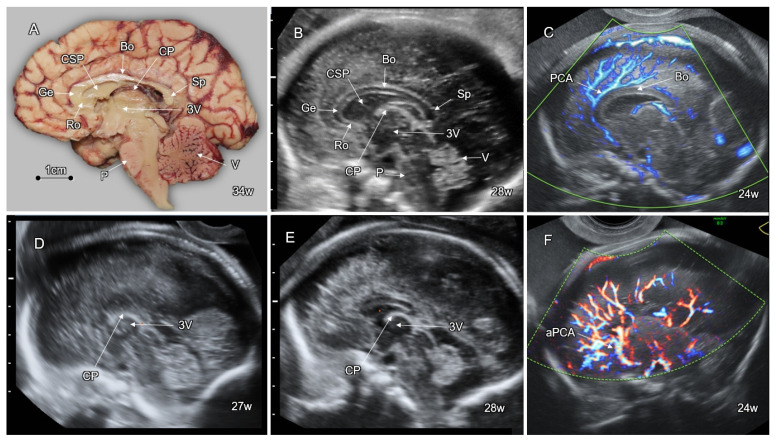
**Fetal corpus callosum in the mid-sagittal plane: normal structure and callosal agenesis. Legend:** (**A**) Median photograph of the brain specimen at 34 weeks, demonstrating the normal structural segments of the corpus callosum: the rostrum (Ro), genu (Ge), body (Bo), and splenium (Sp). Note the cavum septi pellucidi (CSP) below the corpus. 3V, the third ventricle; P, the pons; and V, the vermis. (**B**–**E**) Mid-sagittal sonographic fetal head planes: (**B**) A normal fetus at 28 weeks of gestation. Note the anatomical structures indicated in panel A. CP, the choroid plexus of the third ventricle. (**C**) A normal fetus at 24 gestational weeks. The regular path of the pericallosal artery (PCA), applying high-definition Doppler, is shown. (**D**) A fetus at 27 gestational weeks with complete agenesis of the corpus callosum. (**E**) A fetus at 28 gestational weeks with partial agenesis of the corpus callosum; note the lack of the rostrum, genu, and splenium. (**F**) A fetus with complete agenesis of the corpus callosum at 24 gestational weeks. Note an abnormal branching of the pericallosal artery (aPCA) demonstrated by high-definition Doppler.

The corpus callosum (CC) is the largest brain commissure, consisting of approximately 200 million axons. Agenesis of the corpus callosum (ACC) is one of the most common congenital brain anomalies, with an incidence of 1.8/10,000 in the general population and 230–600/10,000 in children with neurodevelopmental disabilities [70]. The basic structural parts of the CC (rostrum, genu, body, and splenium) are formed by mid-gestation (Figure 12A). It reaches full maturity only in adolescence, with typical thinning of the isthmic segment between the body and the splenium. The axons of the different parts connect distinctive cortical regions: the rostrum—the fronto-basal; the genu—the prefrontal and anterior cingulate; the body—the precentral, insular, and posterior cingulate; the isthmus—the precentral, postcentral, and auditory; and the splenium—the posterior-parietal, medial-occipital, and medial-temporal.

The development of the corpus callosum is closely related to the formation of the massa commissuralis by 6 gestational weeks in the dorsal part of the primitive lamina terminalis, which corresponds to the most anterior closure site of the neural tube. By the early second trimester, this region appears as the midline zipper zone where glial cells focally connect between the cortico-septal areas of the developing hemispheres. This zone serves as a substrate for the axons arising from the hemispheres to cross the midline and form the corpus callosum. Abnormal callosal development may be caused by defects in neuronal and glial proliferation, midline patterning, neuronal migration and specification, axonal growth and guidance, and post-guidance development [71]. After crossing the midline, the callosal axons arrive mainly to homotopic contralateral regions [72]. The fetal CC elongates antero-posteriorly in a bidirectional pattern, with the rostrum and the splenium being the last to form [73].

Disorders of the CC present as complete or partial callosal agenesis (Figure 12D,F), hypoplasia (complete and thin CC), hyperplasia (complete and thick CC), or dysgenesis (abnormally shaped CC) [74].

A variety of genetic, metabolic, disruptive, and toxic causes can result in abnormal CC development; however, the etiology of DCC remains unknown in many of the cases [75].

There are more than a thousand different syndromes and metabolic disorders associated with DCC, most of them leading to moderate to severe neurodevelopmental disability [76].

Some examples:

X-linked lissencephaly with ACC and ambiguous genitalia is caused by variants in the *ARX* gene and affects primarily males. Females with ARX variants may have clinical symptoms ranging from none to mental retardation, seizures, and neuropsychiatric disorders. Their MRI is either normal or shows isolated ACC.

Mutations in the *L1CAM* gene cause the X-linked CRASH syndrome, manifesting with ACC, mental retardation, adducted thumbs, spastic paraplegia, and hydrocephalus.

Andermann syndrome is an autosomal recessive condition that is caused by mutations in the *KCC3* gene, and presents with DCC, cognitive impairment, episodes of psychosis, and progressive central and peripheral neuropathy.

De novo *ZEB2* dominant mutations lead to Mowat–Wilson syndrome, in which callosal agenesis is associated with microcephaly, congenital heart defects, Hirschsprung disease, epilepsy, and cognitive impairment.

Aicardi syndrome is believed to be due to de novo mutations in an unknown gene on the X chromosome, affecting only females and males with Klinefelter syndrome. The classic triad of this syndrome includes ACC, chorio-retinal lacunae, and intractable infantile spasms. Patients with Aicardi syndrome may show additional cerebral and ophthalmological abnormalities such as cortical malformations (mostly polymicrogyria), periventricular and subcortical heterotopia, interhemispheric cysts, optic disc coloboma or hypoplasia, vertebral and rib abnormalities, and vascular malformations [72].

Recessive mutations in the *KIF7* and *GLI3* genes are responsible for acrocallosal syndrome, which is characterized by ACC, polydactyly, craniofacial anomalies, and mental retardation [77].

The *TUBA1A* autosomal dominant tubulinopathy manifests with heterogeneous brain malformations (DCC, malformations of cortical development, abnormal basal ganglia, brainstem, and cerebellar anomalies), microcephaly, developmental delay, and epilepsy.

Smith–Lemli–Opitz syndrome is an autosomal recessive disorder caused by mutations in the *DHCR7* gene that results in an inborn error of cholesterol synthesis. Features of this syndrome (growth failure, intellectual disability, behavioral problems, autism, and syndactyly of the second and third toes) may include ACC [78].

Variants in the *FOXG1* gene lead to a peculiar phenotype characterized by callosal dysgenesis and delayed myelinization. Children with this rare autosomal dominant disorder suffer from profound neurodevelopmental delay, hypotonia, motor disorders, gastroesophageal reflux, and microcephaly [79].

ACC is among the most frequent CNS malformations in oral–facial–digital syndrome type-1, caused by *OFD1* variants [74].

**Figure 13:**
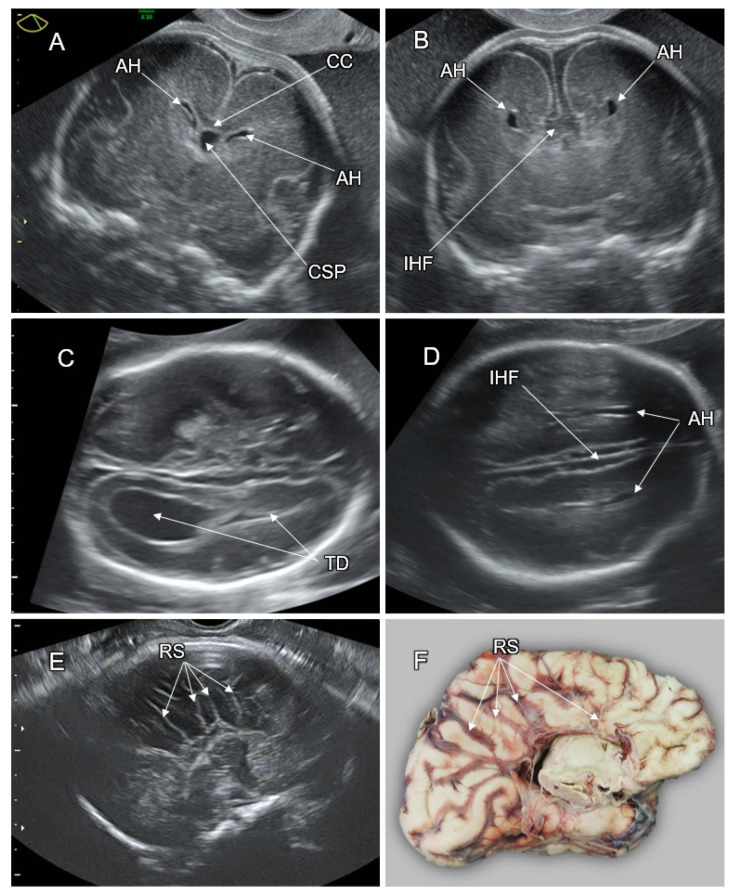
**CNS imaging features of abnormal callosal development. Legend**: (**A**,**B**) Transthalamic coronal planes: (**A**) A normal fetus at 24 gestational weeks. Note the normal oblique alignment of the anterior horns (AH) relative to the corpus callosum (CC), appearing as a concave hypoechoic stripe above the cavum septi pellucidi (CSP). (**B**) A fetus with complete callosal agenesis at 22 gestational weeks. Note the vertically oriented and remote anterior horns, the absence of the CSP, and the wide interhemispheric fissure (IHF). (**C**,**D**) Axial transventricular head planes of a fetus with complete callosal agenesis at 27 gestational weeks. Note the colpocephalic configuration of the lateral ventricle, the “tear drop” sign (TD), remote and parallelly oriented anterior horns (AH), ventriculomegaly, and wide interhemispheric fissure (IHF). (**E**) Sagittal head plane of a fetus with complete callosal agenesis (pACC) at 39 gestational weeks. Note the abnormal radial sulci (RS) converging towards the 3rd ventricle and a lack of the cingulate sulcus (the “sunray” sign). (**F**) Median photograph of the brain specimen at 34 weeks. Note the abnormal radial sulci (RS) converging towards the 3rd ventricle and a lack of the cingulate sulcus (the “sunray” sign).

Pericallosal lipomas can interfere with the normal development of the corpus callosum [80]. Two major types of lipomas are known: tubulonodular and curvilinear. They typically appear as echogenic findings surrounding a short CC, optimally depicted in the coronal and sagittal planes (Figure 13E). Callosal abnormalities due to pericallosal lipoma generally have a better prognosis compared to etiologies of DCC. Curvilinear lipomas are associated with a lower risk of abnormal neurodevelopment than the tubulonodular ones [81].

In utero alcohol exposure decreases gliogenesis and impairs glial–neuronal interactions, which are critical for normal CC formation. DCC can be depicted on prenatal and postnatal neuroimaging in fetal alcohol syndrome [72].

Congenital infection with cytomegalovirus, toxoplasmosis, rubella, and Zika virus have been reported to result in abnormal callosal development in association with additional CNS and systemic findings of intrauterine infection [82,83].

High-resolution fetal neurosonography performed at the second half of gestation enables accurate imaging of the fetal CC. Callosal anatomy can be optimally demonstrated in the mid-sagittal plane, depicting the CC as a convex elongated hypoechoic stripe clearly demarcated between the pericallosal sulci and the CSP (Figure 12B). Color Doppler imaging of the pericallosal artery above the CC can improve callosal detection (Figure 12C). Numerous nomograms have been developed for the evaluation of CC growth throughout the gestational weeks. In fetuses with complete ACC, the callosal tissue is completely absent, whereas in partial ACC, the CC is shortened due to missing segments (usually the splenium, the rostrum, and the genu) (Figure 12D,E). ACC is associated with an aberrant orientation of the pericallosal artery (Figure 12C). Typical abnormal mesial sulcation patter can be observed in fetuses with ACC in the third trimester. It appears as a lack of the cingulate sulci with radial sulci converging towards the third ventricle (“sunray” sign, Figure 13E,F) [80].

On the frontal and transthalamic coronal neurosonograms, the CC appears as a concave hypoechoic stripe above the CSP, bridging between the infero-mesial aspects of the cerebral hemispheres. The CC fibers project over the lateral ventricles, resulting in the typical oblique orientation of the anterior horns (Figure 13A). In fetuses with ACC, the axons that were fated to shape the corpus callosum are misrouted and run parallelly along the infero-mesial aspects of the hemispheres, forming the Probst bundles. These bundles result in the remote and vertically orientated frontal horns (“moose head” or “Viking helmet” sign, Figure 13B) [80].

Since the mid-sagittal and coronal head planes are not routinely assessed on the basic fetal CNS examination [84], attention should be paid to the indirect signs of ACC in the regularly assessed axial transthalamic and transventricular planes. These signs include the absent or abnormally shaped CSP (the CSP length to width ratio of <1.5) [85], colpocephalic configuration of the lateral ventricles (widening of the atria and the occipital horns and narrowing of the anterior ones, the “tear drop” sign), remote and parallelly oriented frontal horns (Figure 13C,D), ventriculomegaly, wide interhemispheric fissure, and upward bulging of the third ventricle [80,86].

In approximately 50% of fetuses with ACC, additional CNS abnormalities may be detected. The most common associated anomalies are malformations of cortical development (polymicrogyria, lissencephaly, pachygyria, schizencephaly) and posterior fossa defects [87]. Destructive parenchymal brain lesions have been reported in 10% of ACC fetuses, raising the possibility of an acquired or genetic–metabolic underlying etiology [88]. Associated abnormalities of the Sylvian fissure and the hippocampus have been reported to aggravate the neurodevelopmental outcome [75]. The most common symptoms in individuals with ACC are delayed motor and cognitive functions, social and language deficits, and epilepsy. Autism, schizophrenia, and attention-deficit disorders have also been reported [89].

New genetic techniques (CMA, WES) enable diagnosis of the underlying genetic etiology of DCC. About 10% of patients have chromosomal anomalies and 20–35% are identified with recognizable genetic syndromes [72].

In pregnancies with a diagnosis of fetal ACC, counseling by a multidisciplinary team (neurosonographer, pediatric neurologist, and geneticist) should be offered. Dedicated scanning for associated CNS and systemic malformations, amniocentesis for CMA and WES, and fetal brain MRI should be performed to elucidate underlying genetic abnormalities and accompanying structural anomalies, which can worsen the prognosis. In the presence of additional pathological findings, there is a high probability of syndromic ACC, leading to neurodevelopmental disabilities [76].

In apparently isolated fetal ACC, the risk of additional postnatally detected anomalies is 5% and 15% for complete and partial ACC, correspondingly [74]. According to a meta-analysis by D’Antonio et al. [89], a normal neurodevelopmental outcome is expected in 76.0% (95% CI 64.3–86.1%) and 71.4% (95% CI 53.1–86.7%) of children with complete and partial prenatally diagnosed isolated ACC, respectively. A fetal brain MRI anatomical score based on evaluation of gyration, opercularization, temporal lobe symmetry, parenchymal lamination, hippocampal position, basal ganglia, and ventricular size has recently been developed to improve prognostication of postnatal neurodevelopmental outcome [75].

## 6. Malformations of Cortical Development

The cerebral cortex constitutes the outer surface of the brain. The neocortex has a six-layered cellular structure of 2–5 mm width. Programmed cortical expansion and folding play a key role in the development of the human brain. Abnormal cortical formation can lead to inadequate intellectual performance and neurological disorders. Cortical development is governed by three overlapping processes: neuronal and glial proliferation, neuronal migration, and post-migration neuronal development and organization (see Section 2) [90].

Malformations of cortical development (MCD) are a heterogeneous group of malformations related to disorders in one or more of these processes [91]. MCD are an important cause of epilepsy, developmental delay, and motor and sensory deficits. Over 75% of MCD patients develop seizures during their lives, and over 40% of pediatric MCD cases suffer from therapeutically refractory seizures. MCD are associated with cognitive impairment and autism [92]. Genetic factors play an important role in MCD etiology. Different mutations in the same gene can result in phenotypes of variable severity, according to the degree of dysfunction of the mutant protein. Intrauterine infections, vascular injury, trauma, and exposure to teratogens can also cause malformations of cortical development [93]. MCD are classified according to the earliest regulating process affecting cortical development [91].

### 6.1. MCD Secondary to Abnormal Neurogenesis

Abnormal neurogenesis refers to decreased or increased proliferation or apoptosis of neurons and glia. Disorders of these processes result in a deficient or excessive population of neural cells, leading to primary microcephaly or congenital megalencephaly, correspondingly.

#### 6.1.1. Microcephaly

Microcephaly (MIC), a too-small head, is frequently associated with cognitive deficits and neurological abnormalities. Primary microcephaly results from decreased proliferation or increased apoptosis of neural and glial cells. Babies with isolated primary MIC are typically born with a pathologically small HC. Genes responsible for primary MIC include *MCPH1*, *ASPM*, *CDK5RAP2*, *CENPJ*, *STIL*, *WDR62*, *CEP152*, and *CEP63*. All of them encode proteins which regulate centrosome function in the mitotic division of neural and glial cells and have autosomal recessive inheritance [94].

Secondary microcephaly may develop in the post-neurogenesis period due to disruptive events such as ischemia, infection, and trauma, inborn errors of metabolism, teratogens, and genetic disorders. It usually develops during the first two years of life. Secondary MIC may manifest at late gestation if the pathogenic insult occurred early in pregnancy. Deceleration of fetal HC growth in the third trimester can be the only prenatal characteristic of an evolving secondary MIC [95].

Postnatal MIC is defined as a head circumference (HC) more than 2 standard deviations (SD) below the mean for age and gender [96]. It is measured with a centimeter tape, including the scalp and hair.

Prenatal MIC is defined as a fetal HC over 3 SD below the mean for gestational age [97,98]. It is measured by ultrasound around the outer borders of the fetal skull in the transthalamic axial head plane. The difference in methodology between prenatal and postnatal HC evaluation reduces the accuracy of fetal MIC diagnosis. The positive prediction of microcephaly at birth based on sonographic HC evaluation in utero ranges between 57% and 67% [99].

Workup of fetal microcephaly includes confirmation of gestational age, information regarding family history of MIC and intellectual disability and parental consanguinity, measurement of parental HC, dedicated neurosonography, brain MRI, maternal serological tests for TORCH infection (Zika virus in endemic regions), detailed ultrasound scan for fetal growth and anatomy, and amniocentesis for CMA and WES [95]. The measurement of the foramen magnum-to-cranium distance for evaluation of fetal head growth in the vertical dimension can improve the accuracy of fetal MIC diagnosis [100].

In newborns, the degree of HC smallness (expressed as the number of SD below the mean for gestational week at birth and sex) correlates with the occurrence of intellectual disability. For cut-offs of –2 SD and –3 SD, respectively, approximately 10% and 50% of MIC babies will have cognitive impairment [101]. The data on the correlation between the severity of prenatal MIC and the prevalence of neurodevelopmental disability after birth are very limited, making prognosis for fetal MIC difficult [95].

#### 6.1.2. Megalencephaly

Megalencephaly refers to brain overgrowth due to excessive proliferation or decreased apoptosis of neural and glial cells. Macrocephaly is defined as an HC that is at least 2 SD above the mean in both pre- and post-natal evaluations.

It is rare to diagnose syndromic megalencephaly in utero. In most cases, megalencephaly manifests postnatally. Syndromic megalencephaly may be associated with overgrowth syndromes (Sotos, Weaver, Simpson–Golabi–Behmel), megalencephaly-capillary malformation (MCM), megalencephaly–polymicrogyria–polydactyly–hydrocephalus (MPPH), and hemimegalencephaly. Numerous genes associated with abnormal PI3K-AKT-mTOR pathway activation cause most of these syndromes [102]. Autism, developmental delay, intellectual disability, or early-onset seizures are associated with syndromic megalencephaly [103].

Prenatally diagnosed macrocephaly is usually familial, isolated, and benign. Several disorders other than megalencephaly can cause macrocephaly, including hydrocephalus and enlargement of the subarachnoid spaces, brain tumor, and massive subdural hemorrhage. Macrocephaly can accompany skeletal dysplasias (achondroplasia, thanatophoric dysplasia, Apert syndrome) and metabolic disorders (Canavan syndrome, glutaric aciduria).

### 6.2. MCD Secondary to Abnormal Neuronal Migration

MCD secondary to abnormal neuronal migration primarily refer to lissencephaly, cobblestone malformation, and gray matter heterotopia.

#### 6.2.1. Lissencephaly

The term “lissencephaly” is derived from the Greek words *lissos*, meaning “smooth” and *enkephalos*, meaning “brain”. Lissencephaly (formerly defined as lissencephaly type-1 or classical lissencephaly) is caused by neuronal under-migration. In lissencephaly, the cerebral cortex is abnormally thick, composed of two or four cellular layers, and the gyration is absent or reduced (agyria or oligogyria) [93,104]. The term pachygyria is also used to indicate a few broad gyri and shallow sulci. Mutations of multiple genes are responsible for lissencephaly. Most of them are related to tubulin and microtubule-associated proteins [105]. The MRI classification of lissencephaly is based on the grade of cortical smoothness, the gradient of the gyration, cortical thickness, and associated brain abnormalities. This classification allows for the linkage of various lissencephaly phenotypes to certain genotypes [106].

**Figure 14:**
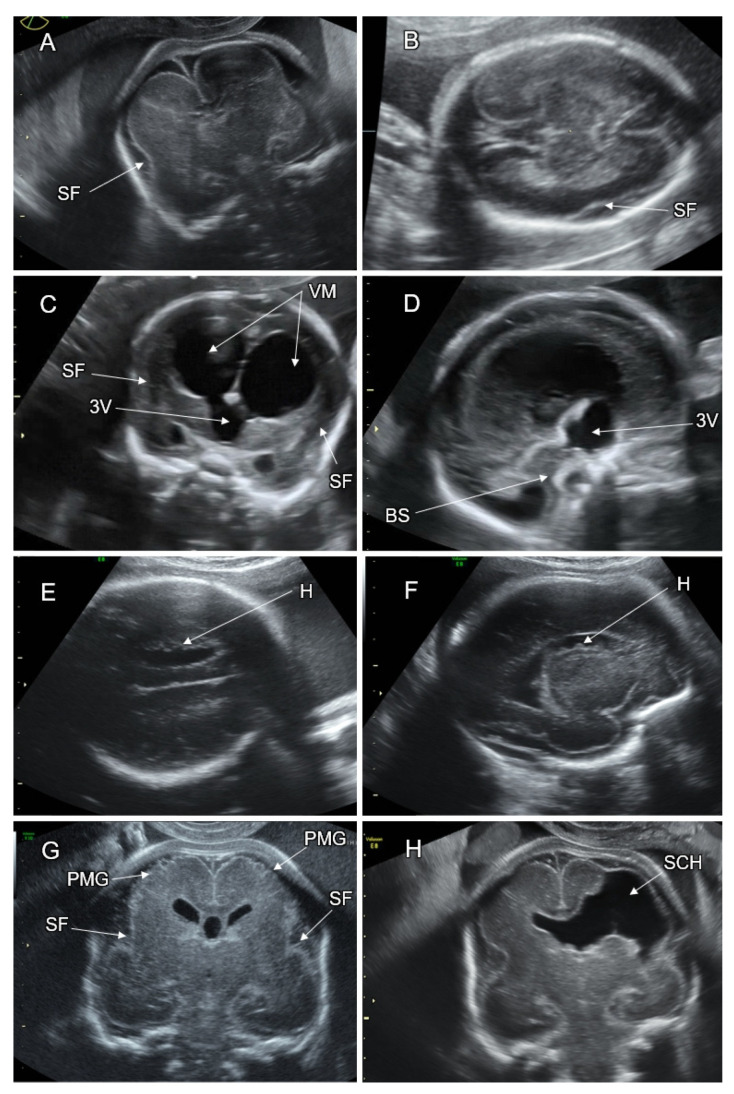
**Fetal sonographic features of malformations of cortical development. Legend:** (**A**,**B**) Transthalamic coronal and axal planes of a fetus with lissencephaly at 24 gestational weeks. Note the shallow Sylvian fissures (SF). (**C**,**D**) Transventricular axial and sagittal planes of a fetus with Walker–Warburg syndrome at 26 gestational weeks. Note the flat Sylvian fissures (SF), severe dilatation of the lateral (VM) and third (3V) ventricles, and the typical Z-shaped hypoplastic brainstem (BS). (**E**,**F**) Transventricular axial and sagittal ventricular planes of a female fetus with periventricular nodular heterotopia at 32 gestational weeks. Note the chain of the small subependymal protrusions bulging into the ventricle (PNH). (**G**) Transthalamic coronal plane of a fetus with bilateral frontal polymicrogyria at 25 gestational weeks. Note an irregular, serrated cortical contour (PMG). (**H**) Transthalamic coronal planes of a fetus with schizencephaly at 22 gestational weeks. Note the extensive lateral parenchymal cleft (SCH) extending from the cerebral cortex to the ventricle associated with the absence of the cavim septi pellucidi.

Mutations in the genes of lissencephaly can be sporadic autosomal dominant (de novo), autosomal recessive, or X-linked. The *LIS1* gene variant on 17p3 was the first to be identified. *LIS1* is missing due to a 17p3 deletion in patients with Miller–Dieker syndrome, which is also characterized by a specific facial dysmorphism. On late second and third trimester neurosonography, lissencephaly manifests with a bilaterally reduced or absent sulcation (Figure 14A,B). The fetal brain has a typical figure “8” shape in the coronal transthalamic plane. *LIS1* lissencephaly is characterized by a lack of occipital gyration and a relatively spared frontal gyration (a posterior to anterior gradient).

Lissencephaly with an anterior to posterior gradient favors X-linked *DCX* gene variants. In lissencephaly with an anterior to posterior gradient and severe cerebellar hypoplasia, mutations of *RELN*, *CDK5*, or *VLDLR* should be suspected, whereas a posterior to anterior gradient with agenesis of the corpus callosum is indicative of *ARX* or *TUBA1A* mutations [106].

A fetal brain MRI should be performed to evaluate CNS anomalies that can be associated with lissencephaly. Detection of subcortical band heterotopia, hydrocephalus, ACC, or posterior fossa anomalies can provide clues to possible genetic causes. CMA and WES studies are needed to clarify the diagnosis.

Patients with lissencephaly typically have intractable seizures and profound intellectual disability [107].

#### 6.2.2. Cobblestone Malformation (CM)

CM, formerly classified as lissencephaly type-2, is caused by neuronal and glial over-migration out of the pial lining. The cortical surface in CM has a characteristic bumpy, granular appearance, lacking normal sulcation. This malformation is related to abnormal O-glycosylation of α-dystroglycan on the end-feet of radial glia cells, which is required for proper interaction with the pial basement membrane and prevention of neuronal over-migration [24]. Mutations in *POMT1*, *POMT2*, *POMGnT1*, *FKTN*, *FKRP*, *LARGE*, *B3GALNT2*, *B3GNT1*, *GALNT2*, *GTDC2*, *ISPD*, and *TMEM5* lead to a spectrum of autosomal recessive disorders (α-dystroglycanopathies) that may manifest with cerebral, ocular, and muscular abnormalities (Walker–Warburg syndrome, muscle–eye–brain syndrome, and Fukuyama muscular dystrophy) [91].

The most severe type of CM is Walker–Warburg syndrome. On neuroimaging, the cerebral parenchyma is thin, and the cortex is mildly thickened, depicting radial striation and a serrated interface between the gray and white matter. Severe ventriculomegaly, cerebellar and vermian hypoplasia, and a “Z”-shaped hypoplastic brainstem are consistent features of Walker–Warburg syndrome [104] (Figure 14C,D). Cephalocele, retinal dysplasia, microphthalmia, and congenital muscular dystrophy can be associated with this syndrome. The median survival is less than 1 year. All survivors have profound mental retardation [108].

#### 6.2.3. Gray Matter Heterotopia

Gray matter heterotopia refers to clusters of normal neuronal cells abnormally located in the periventricular zones, the white matter, or the outer cortical margins, all related to impaired migration. Periventricular nodular heterotopia (PNH) is the most common type. Subcortical heterotopia, in which neuron clumps are found within the white matter, is rarer. On neuroimaging, PNH appears as nodular bulges into the ventricle (Figure 14E,F). PNH can appear as a single bulge or scattered findings. Single PNH may be an incidental benign finding, be caused by focal ependymal disruptions (i.e., hydrocephalus), or be associated with genetic syndromes (i.e., Smith–Magenis). Scattered PNH is typical of *FLNA* mutations, affecting only female patients, in which PNH presents with a specific “string of pearls” sign and an enlarged cisterna magna. Pathogenic mutations in the *ARFGEF2* gene present as PNH associated with congenital microcephaly, implying a poor prognosis [109].

### 6.3. MCD Due to Abnormal Post-Migration Neuronal Development and Cortical Organization

MCD secondary to abnormal post-migration neuronal development and cortical organization result in polymicrogyria and cortical dysplasia. These anomalies develop in the late second and third trimesters of pregnancy, affecting the formation of the six-layered cortex, outgrowth of axons and dendrites from cortical neurons, and synaptogenesis.

#### 6.3.1. Polymicrogyria

Polymicrogyria (PMG) is characterized by an excessive number of small gyri separated by shallow sulci, giving the cortex an irregular, bumpy surface with apparent thickening, an irregular gray–white matter interface, and a prominent subarachnoid space overlying the affected cortex. PMG can be focal, diffuse, unilateral, or bilateral. On prenatal neuroimaging, PMG appears as a focal or diffuse cortical irregularity or multiple small infoldings of the affected cortex (Figure 14G). Fetal PMG most frequently affects the Sylvian fissure depicting an abnormal opercular contour.

PMG has a multifactorial pathogenesis including chromosomal abnormalities, single gene mutations, intrauterine infection, exposure to teratogens, and damage due to ischemia or vascular disruption [110].

Congenital CMV infection is the most common cause of fetal PMG. It may be associated with ventriculomegaly, ventricular wall irregularity, periventricular pseudocysts (specifically in the temporal and occipital horns), ventricular adhesions, abnormal periventricular echogenicity, parenchymal calcifications, cerebellar and callosal anomalies, microcephaly, and intracranial hemorrhage due to fetal thrombocytopenia.

The most common CMA abnormalities associated with PMG are 22q11.2 deletion and 1p36 deletion. Mutations of more than 40 genes have been related to PMG, including genes of the PI3K-AKT-MTOR pathway, tubulinopathies, and α-dystroglycanopathies [109].

In patients with PMG, the prevalence of epilepsy and global developmental delay is high, reaching 78% and 70%, correspondingly [111].

#### 6.3.2. PMG Associated with Schizencephaly

PMG typically occurs in the cortical lining of schizencephaly. This malformation is defined as a parenchymal brain cleft extending from the cerebral cortex to the ventricle. It is believed to be a result of an early disruptive injury to the developing cerebral hemispheres. The most common type of parenchymal cleft correlates with the distribution of the middle cerebral artery. It is typically associated with the absence of the cavum septi pellucidi (Figure 14H). Schizencephaly is classified as type I (closed lip) or type II (open lip). In type II, the missing parenchyma is replaced by CSF and can be clearly demonstrated on a prenatal brain scan. In type I, the lips of the cleft face each other closely, making detection difficult [104]. The neurologic manifestations of schizencephaly depend on the type and location of the lesion. Patients with type I lesions usually manifest with epilepsy, while patients with type II present with severe developmental disabilities, microcephaly, and intractable seizures [112].

The neurosonographic signs suggestive of fetal MCD include abnormalities of the Sylvian fissure, delayed cortical sulcation, prematurely appearing or aberrant sulci, ventricular wall irregularity, abnormal parenchymal thickness, abnormal shape and orientation of the sulci and gyri, hemispheric asymmetry, simplified gyral pattern, cortical cleft, and parenchymal echogenicities. Pregnant women with family history of MCD, cases of intrauterine infection, and fetuses with ventriculomegaly, abnormal head circumference, callosal disorders, cerebellar, vermian, or brainstem abnormalities should be evaluated for MCD. When fetal MCD is suspected, a dedicated neurosonography and a detailed systemic anatomical scan should be performed. Serological tests for intrauterine infection, fetal head MRI, and genetic studies for CMA and WES are recommended. Parents should be referred for multidisciplinary counseling by specialists in pediatric neurology, genetics, neurosonography, and MRI [109].

## 7. Malformations of the Posterior Fossa

The posterior fossa (PF) contains the brainstem, cerebellum, and surrounding CSF spaces. The tentorium separates the PF structures from the occipital lobes. The upper part of the brainstem (the midbrain) is derived from the mesencephalon, while the pons, cerebellum, and medulla oblongata develop from the rhombencephalon. Malformations of the brainstem and cerebellum are frequently associated with supratentorial anomalies. They can be the only detectable abnormality in patients with intellectual disability or autism.

The mechanisms underlying PF anomalies are variable. They include abnormal anteroposterior patterning of the mesencephalon and rhombencephalon, resulting in rostral malposition of the isthmus organizer (described in Opitz G/BBB syndrome); mesenchymal–neuroepithelial signaling defects (manifesting as Dandy–Walker malformation complex); and numerous genetic variants responsible for disorders of the Reelin pathway, α-dystroglycanopathies, ciliopathies, and tubulinopathies in which PF anomalies are associated with cerebral malformations. They may also be caused by teratogens, infections, and vascular disruption. PF anomalies have heterogeneous imaging features and a wide spectrum of neurologic manifestations [113,114].

Development of the cerebellar folia and vermian lobulation can be reliably assessed on prenatal neuroimaging only by late second trimester. Therefore, many PF malformations cannot be correctly diagnosed earlier [115].

Classification schemes of PF malformations are based on embryological, genetic, neuroimaging, and clinical criteria [113,114].

A practical approach to prenatal diagnosis of PF malformations using MRI was proposed by Guibaud in 2004 [116]. This approach implemented the classification of PF malformations into three groups according to the major imaging abnormality: (1) increased PF fluid-filled CSF space, (2) decreased cerebellar biometry, and (3) focal lesion of the cerebellum. In 2010, Garel developed a diagnostic workup for fetal PF malformations that used the mid-sagittal and axial transcerebellar head planes with either neurosonography or MRI [117]. According to this workup, fetal PF malformations can be characterized by addressing the following features: (1) the size of the PF fluid spaces; (2) the position and orientation of the tentorium; (3) cerebellar biometry; (4) cerebellar and brainstem morphology; (5) fourth ventricle morphology; and (6) cerebellar echogenicity or MRI signal.

A few common examples of PF malformations:

### 7.1. Dandy–Walker Malformation (DWM)

**Figure 15:**
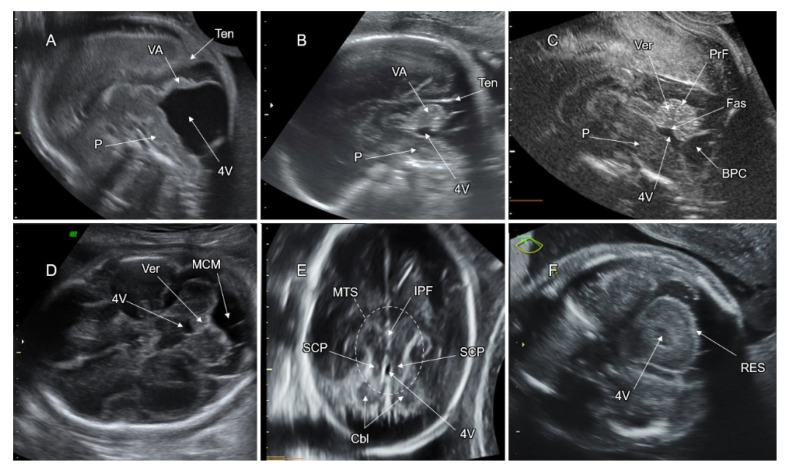
**Posterior fossa malformations. Legends:** (**A**–**C**) Mid-sagittal head planes: (**A**) A fetus at 22 weeks of gestation with Dandy–Walker malformation. Note the enlarged posterior fossa with upward displacement of the tentorium cerebelli (Ten), cystic dilatation of the 4th ventricle (4V) that fills the entire posterior fossa, and almost complete agenesis and substantial upward rotation of the vermis (VA). (**B**) A fetus at 28 gestational weeks with inferior vermian agenesis. Note the small vermis with mild upward rotation, lacking all the inferior lobules (VA). The tentorium (Ten) is in a normal position. The fastigium and the primary vermian fissure are not formed (compared with a normal vermian structure in panel (**C**). (**C**) A fetus at 25 gestational weeks with a Blake’s pouch cyst (BPC). Note the wall of the cyst, protruding from the floor of the fourth ventricle into the cisterna magna. The vermis is of normal size and shape and only slightly rotated upwards. Note the normal fastigial beak (Fas) in the middle of the intraventricular vermian contour and the normally located primary vermian fissure (PrF) dividing between the upper and lower vermian regions in a 1:2 ratio. (**D**–**F**) Axial transcerebellar head planes: (**D**) A fetus at 27 gestational weeks with megacisterna magna (MCM). Note the vermian tissue that completely separates between the normally shaped 4th ventricle (4V) and the symmetrically enlarged cisterna magna (MCM). (**E**) Axial plane through the upper cerebellar region of a fetus at 25 gestational weeks with Joubert syndrome. Note the absence of the vermis and the elongated shape of the 4th ventricle (4V) (compared with normal anatomy in panel (**C**). Note the “molar tooth sign” (MTS) encircled by a dashed ellipse, depicting the typically elongated and thickened superior cerebellar peduncles (SCP) running on both sides of the 4th ventricle (4V) and the deep interpeduncular fossa (IPF). (**F**) A fetus at 19 gestational weeks with rhombencephalosynapsis. Note median fusion of the cerebellar hemispheres (Cbl), resulting in a small and round-shaped cerebellum lacking the vermian tissue and the posterior cerebellar notch (RES).

The diagnosis of DWM is based on three imaging features: (1) an enlarged posterior fossa with upward displacement of the tentorium cerebelli; (2) cystic dilatation of the fourth ventricle that fills nearly the entire retrocerebellar space; and (3) partial or complete vermian agenesis and substantial upward rotation of the vermis [116] (Figure 15A).

DWM occurs in 1:25,000–1:35,000 births. Associated chromosomal abnormalities (mainly trisomy 18 and 13) can be detected in as many as 35% of cases. In 30–50% of cases, DWM accompanies other CNS anomalies, including callosal agenesis, occipital encephalocele, polymicrogyria, gray matter heterotopias, and holoprosencephaly. Associated systemic anomalies include facial clefts, congenital heart defects, urinary malformations, and polydactyly. DWM occurs in numerous syndromes (Walker–Warburg, Meckel–Gruber, Aicardi, Neu–Laxova, and more). Mutations in *FOXC1*, which is specifically expressed in the mesenchyme overlying the cerebellum, are an established cause of DWM [113,116].

The differential diagnosis of DWM includes other conditions leading to cystic dilatation of the posterior fossa: Blake’s pouch cyst, mega cisterna magna, PF arachnoid cyst, and destruction of cerebellar tissue after a clastic insult [114]. When DWM is associated with additional CNS malformations, the neurodevelopmental outcome is usually poor. In fetuses with isolated DWM and a well-formed vermis, the prognosis is better [118].

### 7.2. Vermian Agenesis (VA), Hypoplasia (VH), and Dysgenesis (VD)

VA refers to the complete or partial absence of vermian tissue. In partial VA, the missing part is typically the inferior one due to cranio-caudal development of the vermis [116]. VH refers to a small but completely formed vermis without missing lobules. Differentiation between VH and VA in utero is difficult due to imaging limitations. On postnatal MRI, VH is frequently described with abnormal vermian lobulation and a dysmorphic fastigial shape [114]; the term “vermian dysgenesis” is more appropriate for description of such findings [119]. Despite the difference in definitions, the terms “VA”, VD”, and “VH” are interchangeably used in the literature [120].

Demonstration of a continuity between the fourth ventricle and the cisterna magna on an axial fetal head scan raises the possibility of VA. To confirm VA diagnosis, a precise mid-sagittal plane oriented to the PF, preferably through the posterior fontanel, should be used [84]. In fetuses with inferior VA, the vermis is small and upwardly rotated, although to a lesser extent than in DWM. The inferior vermian lobules are missing, resulting in a flat fastigial shape and an abnormal division of the vermis by the primary fissure. A normal position of the tentorium helps in differentiating VA from DWM (Figure 15B). It should be noted that on neurosonography of fetuses with VA, even a slight deviation of the scanning plane from the midline may mimic a “normal vermis” caused by insonation through the mesial part of the adjacent cerebellar hemisphere. MRI is helpful for evaluation of VA as well as for assessment of associated CNS malformations [116,118].

Due to late vermian maturation, the diagnosis of vermian abnormalities can remain unclear prior to 24 weeks. A detailed neurosonographic examination of the vermis should be performed, including assessment of the fastigium, the primary fissure, and vermian biometry on the mid-sagittal plane [118].

Inferior VA and VD may be associated with intellectual disability and autistic spectrum disorder. Information regarding abnormal neurodevelopmental outcomes in children with a prenatal diagnosis of vermian anomalies is limited. Inaccuracy of prognostication can also be caused by inadequate differentiation between VA, VD, and VH in utero. According to a recent meta-analysis, the mean rate of abnormal neurodevelopmental outcomes after prenatal diagnosis of isolated VH was 30.7%; however, it was not statistically significant due to the very small number of the studied cases [121].

### 7.3. Blake’s Pouch Cyst (BPC)

Persistence of BPC is thought to be due to delayed fenestration of the posterior membranous area of the developing fourth ventricle. The walls of the cyst can be demonstrated on axial and sagittal planes, protruding from the floor of the fourth ventricle into the cisterna magna. The BPC contains anechoic CSF, contrasting with the surrounding subarachnoid space. In the mid-sagittal plane, the vermis has normal morphology and size and is only slightly upward rotated (Figure 15C). Occasionally, the inferior vermis may be compressed, resulting in mild fastigial flattening. The fourth ventricle is continuous with the inner space of the cyst. Measurement of the brainstem–vermis angle has been proposed for differentiating between BPC, inferior VA, and DWM. In cases with BPC, the angle is less than 30°, in DWM it is more than 45°, and in inferior VA the angle has intermediate values [122]. The risk of abnormal neurodevelopment following prenatal diagnosis of isolated BPC is low (4.7%) and the risk of chromosomal anomalies is 5.2%. An association with additional CNS anomalies after birth has not been found [121].

### 7.4. Mega Cisterna Magna (MCM)

MCM refers to a symmetrically enlarged cisterna magna of more than 10 mm in the anteroposterior dimension, measured on the axial transcerebellar plane. Unlike DWM, VH, and BPC, there is no continuity between the fourth ventricle and the cisterna magna in MCM. (Figure 15D). The risk of abnormal neurodevelopmental outcome following prenatal diagnosis of isolated MCM is 13.8%. There is no association with chromosomal or CNS anomalies after birth [121].

### 7.5. Joubert Syndrome (JS) and JS-Related Disorders

This group of rare disorders is caused by abnormal function of the primary nonmotile cilia (ciliopathies). The cilia are implicated in neuronal cell proliferation and axonal guidance. More than 30 ciliopathy genes are known, most of which have autosomal recessive inheritance, with a recurrence risk of 25% [114].

Children with JS present with hypotonia, developmental delay, ataxia, eye movement abnormalities, and abnormal breathing. JS-related disorders are characterized by additional abnormalities such as coloboma, liver fibrosis, tongue tumors, polydactyly, nephronophthisis, retinal dysplasia, and cystic dysplastic kidneys.

On neuroimaging, JS is characterized by VA and the pathognomonic upper brainstem malformation, the “molar tooth sign”. This sign can be observed on the axial plane through the upper cerebellar region due to the deep interpeduncular fossa anteriorly, and the elongated and thickened superior cerebellar peduncles running on both sides of the fourth ventricle posteriorly (Figure 15E). The fourth ventricle has typical dysmorphic features: a horizontally orientated ventricular roof on the sagittal plane and anteroposterior elongation of the ventricular shape on the axial one [123].

Brainstem and supratentorial abnormalities (PMG, ACC, gray matter heterotopia, and occipital cephalocele) may accompany JS in about 30% of cases [114].

### 7.6. Rhombencephalosynapsis (RES)

RES is defined as median fusion of the cerebellar hemispheres, dentate nuclei, and cerebellar peduncles associated with a complete or partial absence of the vermis. The genes responsible for this malformation are unknown. RES is usually a sporadic malformation, but it may be part of Gomez–Lopez–Hernandez syndrome, VACTERL, or holoprosencephaly [124].

In fetuses with RES, the cerebellum is small and round-shaped. The posterior cerebellar notch is absent (Figure 15F). The folia run continuously on the fused cerebellar surface. On the mid-sagittal plane, the vermis is absent, although some vermian tissue can be preserved (partial RES). The primary vermian fissure and the fastigium are absent [114]. Neurodevelopmental outcomes of patients with RES range from normal to severe neurological impairment. RES may be complicated by hydrocephalus. Cases with partial RES associated with other CNS or systemic anomalies may have abnormal neurodevelopment [124].

### 7.7. PF Anomalies Associated with Tubulinopathies

Autosomal dominant mutations in tubulin genes can cause VA, small dysplastic cerebellum, and brainstem abnormalities. Cerebellar dysplasia is characterized by a typical diagonal hemispheric cleft involving the superior vermis. Cerebral features include lissencephaly, callosal dysgenesis, and abnormal basal ganglia. Patients can suffer from intractable epilepsy and intellectual disability, facial dysmorphism. Systemic malformations are rare [114].

### 7.8. PF Anomalies Associated with Cobblestone Malformation (CM)

CM is related to defects in α-dystroglycan glycosylation, which affects linkage of the end feet of radial glial cells with the pial limiting membrane, leading to Walker–Warburg syndrome, muscle–eye–brain disease, and Fukuyama muscular dystrophy [91] (see Section 6). Fetuses with CM may present with a hypoplastic kinked brainstem, small cerebellar hemispheres, a small and dysplastic vermis, and an enlarged dysmorphic tectum overhanging the vermis (Figure 14D). The associated supratentorial anomalies include absent or delayed cortical sulcation, ventriculomegaly, ocular malformations, encephalocele, and corpus callosum dysgenesis [114].

## 8. Ventriculomegaly

Fetal ventriculomegaly (VM) refers to enlargement of the lateral cerebral ventricles without indicating the underlying etiology. VM is defined as an atrial ventricular width of more than 10 mm [125,126]. According to the recent recommendations of the Society for Maternal–Fetal Medicine, VM is classified as mild (10–12 mm), moderate (13–15 mm), or severe (>15 mm) [127]. Mild to moderate VM is a common finding in the second and third trimesters, observable in about 1% of pregnancies [128]. Hydrocephalus refers to excessive accumulation of the CSF in the central nervous system due to obstructed flow, abnormal production, or absorption. Hydrocephalus is usually associated with increased intracranial pressure. The severity of the VM correlates with the risk of abnormal postnatal neurodevelopment, which should be considered in parental counseling.

**Figure 16:**
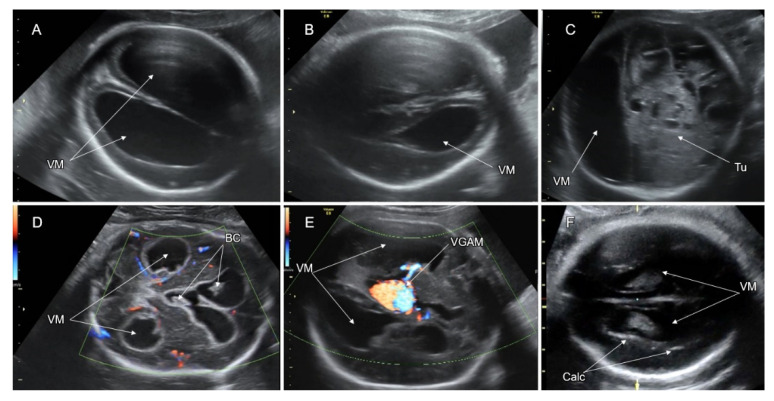
**Fetal ventriculomegaly and associated disorders. Legend:** (**A**–**F**) Fetal head planes on axial sonography: (**A**) A fetus at 31 weeks of gestation with global severe ventriculomegaly (VM) due to aqueductal stenosis. Note the thin and regular ependymal lining; the third ventricle is dilated (not shown). (**B**) A fetus at 29 gestational weeks with complete agenesis of the corpus callosum. Note the colpocephalic dilatation of the atrium and occipital horn (VM). (**C**) A fetus at 26 gestational weeks with a huge intracranial teratoma (Tu). Note a prominent mass effect resulting in midline displacement and severe contralateral ventriculomegaly (VM). (**D**) A fetus at 36 gestational weeks with intraventricular hemorrhage grade-3. Note a thick and irregular ependymal lining, dilated third ventricle, and intraventricular blood clots (BC). (**E**) A fetus at 34 gestational weeks with a vein of Galen aneurysmal malformation (VGAM). Note severe bilateral ventriculomegaly (VM) due to the mass effect of the VGAM, leading to obstruction of the CSF flow from the third ventricle. (**F**) A fetus at 36 gestational weeks with intrauterine CMV infection. Note bilateral ventriculomegaly (VM) and diffuse periventricular calcifications (Calc).

Fetal VM can be caused by obstruction of the CSF circulation within the ventricular system, be secondary to acquired brain damage (intraventricular hemorrhage, ischemic insult, or intrauterine infection), be a result of the mass effect of a brain tumor or cyst, or be associated with CNS anomalies (NTD, disorders of the corpus callosum, malformations of the posterior fossa, or malformations of cortical development) (Figure 16). VM may be part of many genetic syndromes and metabolic disorders and may accompany systemic anomalies [129]. Although an “isolated” fetal VM can be suggested by the exclusion of the underlying disorders, additional anomalies can be detected after birth in 7–10% of fetuses with apparently isolated mild VM [130].

The atrium is the widest part of the lateral ventricle occupied by the glomus of the choroid plexus. The atrial width should be measured in a symmetric transventricular axial plane, clearly demonstrating the atrium and occipital horns. The landmarks of this plane are frontal horns and cavum septi pellucidi (or fornix columns) anteriorly and a fluid-filled ambient cistern posteriorly [126]. The calipers should be positioned on the atrial margins at the level of the parieto-occipital fissure or through the glomus of the choroid plexus and directed perpendicular to the long axis of the lateral ventricle [127]. Optimal atrial visualization is typically obtained for the distant ventricle relative to the ultrasound probe. Measurement of the close ventricle is more difficult because of acoustic shadowing and artefacts. Axial insonation through the mastoid fontanel or scanning on the transcerebellar coronal plane via the posterior fontanel can be used for demonstration of the obscured lateral ventricle [131]. Correct evaluation of the atrial width is crucial for reducing false-positive or false-negative results, particularly in borderline ventricular measurements (about 10 mm) [84].

Ventricular asymmetry is a common finding, defined as a difference in atrial width of more than 2 mm. Fetal VM is unilateral in approximately 50–60% of cases [127]. Ventricular asymmetry without VM is considered a normal variant. Follow-up during later gestational weeks should be taken into account to rule out the development of VM.

Although VM is more common in male (65–75% of cases) than female fetuses, the neurodevelopmental outcome of the ventriculomegaly does not differ by fetal sex [132].

The diagnostic workup for fetal VM includes history taking for neurodevelopmental disorders, dedicated neurosonography, targeted scanning for associated systemic anomalies, fetal echocardiography, maternal serological tests for intrauterine infection (TORCH and Zika virus in endemic areas), amniocentesis for chromosomal microarray and infectious agents (if clinically indicated), and fetal brain MRI [127,132].

Neurosonographic examination of fetal VM should include the morphology and size of the ventricular horns, ventricular asymmetry, contour of the ventricular wall, ependymal thickness and echogenicity, parenchymal periventricular changes (calcifications, cysts, increased echogenicity), and abnormal intraventricular findings (blood clots, septations). The third ventricle, Sylvian aqueduct, and fourth ventricle should be inspected for findings related to obstructive CSF accumulation. Evaluation of cortical sulcation, mid-sagittal brain structures, pericerebral spaces, and posterior fossa anatomy can provide key findings for diagnosis of underlying disorders associated with VM [133].

When CSF flow is blocked below the level of the third ventricle, obstructive VM usually presents with global ventricular dilatation, a large (for gestational age) head circumference, destruction of the septi pellucidi, obliteration of the pericerebral subarachnoid spaces, and a prominent suprapineal recess [133].

Fetal ventriculomegaly secondary to hemorrhagic–ischemic events frequently manifests with unilateral or asymmetric VM. In the acute phase of intraventricular hemorrhage, blood clots can be detected inside the dilated ventricle, and the ventricular wall is echogenic, thick, and irregular. Destruction of the brain parenchyma after periventricular venous hemorrhagic infarction (the most severe form of intraventricular hemorrhage) may result in porencephalic lesions, irregular ventricular contours, and focal dilatation of the affected ventricular region. On the other hand, posthemorrhagic persistent ventricular dilatation and widened subarachnoid spaces may reflect white matter volume loss [134].

Differential diagnoses of unilateral VM include hemimegalencephaly (in which the dilated ventricle belongs to the enlarged malformed hemisphere) and rare cases of unilateral obstruction of the foramen of Monroe [133].

In fetuses with intrauterine infection (TORCH), ventriculomegaly can be associated with a periventricular echogenic halo, periventricular pseudocysts (typically located in the temporal and occipital horns), parenchymal and ependymal calcifications, and intraventricular septations. Infectious CNS damage can also manifest as microcephaly and polymicrogyria [83].

Fetal ventriculomegaly can be a part of congenital CNS malformations (agenesis of the corpus callosum, malformations of cortical development, Dandy–Walker malformation, Chiari-II malformation, rhombencephalosynapsis). Therefore, special attention should be paid to the evaluation of median brain structures (CSP, corpus callosum, brainstem, vermis), sulcation pattern, posterior fossa anatomy, and spine.

In a recent study on 341 fetuses with prenatally diagnosed ventriculomegaly, an abnormal karyotype was reported in 6.2% of the cases (3.1% and 7.5% for mild and severe VM, correspondingly), whereas pathogenic CMA results were detected in an additional 6.7% of the cases with a normal karyotype [135]. In another study of 50 fetuses with VM and a normal karyotype, the CMA abnormalities were found in as many as 26% of the cases (9.5% and 37.9% of isolated and non-isolated VM, respectively) [136].

The diagnostic value of WES in the work-up of fetal ventriculomegaly is currently unknown.

Fetal brain MRI is an important contributing tool for fetal VM assessment, providing additive information in 1–14% of cases (primarily related to MCD, callosal, and vermian anomalies) [127].

Fetuses with VM should be repeatedly evaluated throughout gestation. In the prenatal follow-up, ventriculomegaly may progress in 16% of the cases, and additional CNS anomalies may appear in 13% [132].

The risk of abnormal postnatal neurodevelopment of fetuses with VM is aggravated in the presence of severe VM (more than 15 mm), associated CNS, systemic, or genetic abnormalities, and a progressive trend of ventricular dilatation.

In fetuses with apparently isolated mild VM (10–12 mm), survival is high (93–98%) and the risk of abnormal neurodevelopment is approximately 10%. Isolated moderate VM (13–15 mm) may have a higher risk of adverse outcomes (survival rate ranging from 80–97% and risk of abnormal neurodevelopment ranging from 75–93%) [127].

## 9. Conclusions

Disorders of processes that regulate brain development in utero can cause specific congenital CNS anomalies: neural tube defects (dorsal induction); holoprosencephaly (ventral induction); microcephaly or megalencephaly (neuronal proliferation and apoptosis); lissencephaly, cobblestone malformation, or heterotopia (neuronal migration); and polymicrogyria or cortical dysplasia (cortical organization). Many of these malformations are due to underlying genetic abnormalities and are associated with poor neurodevelopmental outcomes and epilepsy. Prenatal ultrasound screening enables the detection of CNS malformations. However, many of them may not be detected until the third trimester or after birth. Parents facing a prenatal diagnosis of CNS malformations should be referred to a multidisciplinary consultation with specialists in fetal neuroimaging (ultrasound and MRI), fetal neurology, and genetics.

## Figures and Tables

**Table 1:** Timetable of human CNS development.

Developmental Process	Gestational Age (Postmenstrual Weeks)	Main Features	Related Anomalies
**Dorsal induction**	5–7	Formation of the neural tube.	Neural tube defects (anencephaly, cephalocele, spina bifida).
**Ventral induction**	6–9	Division of the prosencephalon into two separate telencephalic vesicles (future cerebral hemispheres), formation of optic vesicles, olfactory bulbs, and corresponding facial structures.	Holoprosencephaly.
**Neuronal/glial proliferation**	Beginning at the 10th week, maximal rate at 17–18 weeks, ending at the late 2nd trimester.	Increase in population of CNS cell. The excessive cells undergo apoptosis.	Microcephaly, megalencephaly, hemimegalencephaly.
**Neuronal migration**	12–20	Movement of neural cells from the subventricular zone towards the outer zones of the developing brain, cortical formation.	Lissencephaly, cobblestone malformation, gray matter heterotopia.
**Post-migration neuronal development and cortical organization**	From 22 weeks to postnatal period.	Cortical maturation, outgrowth of axons and dendrites from cortical neurons, and synaptogenesis.	Polymicrogyria, cortical dysplasia.

**Table 2:** Neural tube defects: associated abnormalities and postnatal outcome.

Type of the NTD *	Risk of Chromosomal Anomalies	Associated Anomalies/Syndromes	Outcome
**Anencephaly**	Low	Cleft lip/palate Omphalocele Heart malformations Limb anomalies Amniotic band syndrome **(Risk of associated anomalies is low)**	Incompatible with life
**Cephalocele**	14–18%	Meckel–Gruber syndrome Amniotic band syndrome Frontonasal dysplasia Walker–Warburg syndrome Fraser syndrome Dyssegmental dwarfism von Voss–Cherstvoy syndrome MIH variant of holoprosencephaly Dandy-Walker malformation **(Risk of associated anomalies is high)**	Childhood mortality: 30% for encephalocele 10–25% for cranial meningocele More than 50% of patients with cephalocele experience at least mild developmental delay.
**Spinal dysraphism**	2–16%	Jarcho–Levin syndrome Cerebrocostomandibular syndrome Neu–Laxova syndrome DiGeorge syndrome SDAM (sacral defect with anterior meningocele) OEIS (omphalocele-exstrophy-imperforate anus-spinal defects) complex) **(Risk of associated anomalies is low)**	OSD ^#^ outcomes: 75–80% early adulthood survival 50%—moderate to severe ambulation deficit 60%—sphincter malfunction 19%—IQ less than 70

* NTD—neural tube defect; ^#^ OSD—open spinal dysraphism.
